# Floral enhancement of arable field margins increases moth abundance and diversity

**DOI:** 10.1007/s10841-023-00469-9

**Published:** 2023-03-23

**Authors:** Dan Blumgart, Marc S. Botham, Rosa Menéndez, James R. Bell

**Affiliations:** 1 Rothamsted Insect Survey, Biointeractions and Crop Protection, Rothamsted Research, West Common, AL5 2JQ Harpenden, UK; 2 UK Centre for Ecology & Hydrology, Maclean Building, Benson Lane, Crowmarsh Gifford, OX10 8BB Wallingford, Oxfordshire UK; 3 Lancaster Environment Centre, Lancaster University, LA1 4YQ Lancaster, UK

**Keywords:** Agri-environment scheme, Agro-ecology, Field margins, Floral resources, Insect conservation, Moth conservation, Nectar resources

## Abstract

Moth populations have declined across large parts of north-western Europe since the mid-20th century due, in part, to agricultural intensification. Agri-environment schemes (AES) are widely implemented across Europe to protect biodiversity in agricultural landscapes. Grass field margins enriched with wildflowers typically out-perform grass-only margins in terms of increasing insect abundance and diversity. However, the effect of wildflower enrichment on moths remains largely unstudied. Here, the relative importance of larval hostplants and nectar resources for adult moths within AES field margins are investigated. Two treatments and a control were compared: (i) a plain grass mix, the control, (ii) a grass mix enriched with only moth-pollinated flowers, and (iii) a grass mix enriched with 13 wildflower species. Abundance, species richness and Shannon diversity were up to 1.4, 1.8 and 3.5 times higher, respectively, in the wildflower treatment compared to plain grass. The difference in diversity between treatments became greater in the second year. There was no difference in total abundance, richness or diversity between the plain grass treatment and grass enriched with moth-pollinated flowers. The increase in abundance and diversity in the wildflower treatment was due primarily to the provision of larval hostplants, with nectar provision playing a smaller role. The relative abundance of species whose larval hostplants included sown wildflowers increased in the second year, suggesting colonisation of the new habitat.

**Implications for insect conservation.**

We show that, at the farm scale, moth diversity can be greatly enhanced and abundance moderately enhanced by sowing diverse wildflower margins, providing these insects with both larval hostplants and floral resources, compared to grass-only margins.

**Supplementary Information:**

The online version contains supplementary material available at 10.1007/s10841-023-00469-9.

## Introduction

Agricultural intensification is a major driver of biodiversity loss in western Europe (Donald et al., 2001, Robinson and Sutherland, 2002) and has been linked to the declines of numerous insect taxa including Lepidoptera (Habel et al., 2019a, Habel et al. 2019b Maes and Van Dyck, 2001). Agri-environment schemes (AES) are widely implemented across Europe with the aim of conserving biodiversity and enhancing ecosystem services and therefore reducing the impact of agricultural intensification (Batáry et al., 2015, Kleijn et al., 2006). Field margin options are popular within AES and have been widely adopted, mainly in central and northern Europe and especially in the UK and Switzerland (Haaland et al., 2011). Such options typically apply to arable land and provide financial incentive for farmers to sow the edges of their fields with grasses and/or forbs rather than crops (DEFRA, 2019). Options typically include wildflower and grass mixes or grass only mixes. The benefits of AES field margin options include the enhancement of farmland biodiversity (Marshall et al., 2006, Vickery et al., 2002), the prevention of soil erosion and the protection of watercourses from agricultural runoff (Marshall and Moonen, 2002). Field margins are also important within the agricultural matrix functioning as dispersal corridors linking fragmented habitat patches (Delattre et al., 2013, Threadgill et al., 2020).

There is a large literature documenting the effect of field margins on agriculturally important insects such as crop-pollinating insects (Carvell et al., 2007, Pywell et al., 2007) and predators of pests (Pfiffner and Wyss, 2004). Wildflower strips are known to be an effective conservation measure for many insect taxa in arable land (Boetzl et al., 2021) and a review by Haaland et al. (2011) found that wildflower strips typically support a greater abundance and diversity of insects compared to plain grass margins. Despite the declines in abundance of moths documented in parts of Europe (Antão et al., 2020, Conrad et al., 2006, Groenendijk and Ellis, 2011), the conservation potential of field margins for this group remains less studied. Moths represent an important source of food, both in their larval and adult form, to many other taxa such as birds and bats (Riccucci et al., 2014, Török et al., 2004). In some contexts, moths are important crop pollinators (Buxton et al., 2022) although in the British landscape, their role as pollinators of wildflowers is more important (Macgregor et al., 2019, Walton et al., 2020). The effect of AES field margins on moth abundance and diversity has been investigated in several studies (Alison et al., 2016, Fuentes-Montemayor et al., 2011, Merckx et al., 2012), with generally positive results. However, these studies all compared AES field margins to non-AES field margins but did not compare different types of AES treatments within field margins. One study that compared different types of AES margin found that diurnal moth abundance and diversity was significantly higher in a grass and wildflower mix compared to a plain grass mix (Alanen et al., 2011), although this study did not include nocturnal moths which make up the majority of species. An unpublished report on a multi-year study from an experimental farm reported that micromoth abundance was higher in AES wildflower margins compared to AES tussocky grass margins (Heard et al., 2012). However, in the peer-reviewed literature, studies examining the effect of floral enhancement of AES field margins appears lacking.

It is known that in several lepidopteran species, fecundity can be increased with the provision of sugar sources (Mevi-Schütz and Erhardt, 2005, Song et al., 2007) and some species will preferentially oviposit on plants that are in flower (Janz et al., 2005, Liu et al., 2010), are producing more nectar (Adjei-Maafo and Wilson, 1983) or are in more nectar-rich areas (Janz, 2005). It follows that it may be possible to enhance the value of field margin habitats for moths through the provision of nectar resources. To test this hypothesis, field margin plots with three seed mixes were sown: grass only, grass plus two moth-pollinated flowers (with nectar provision but low larval hostplant value) and grass plus a diverse mix of wildflowers (of both nectar and hostplant value). Two sampling strategies were used: light-traps and nocturnal transects, the latter of which allowed for the observation of individual behaviours (i.e. nectaring, mating, ovipositing, emerging from pupa). We hypothesised that the provision of nectar and additional hostplants in the grass mixes would enhance the attractiveness and reproductive value of the field margins to many species of moth resulting in a higher local abundance and a higher density of larvae due to preferential oviposition in more nectar-rich and hostplant-rich areas. By dividing moths into their feeding guilds, the two effects of larval hostplant and adult nectar source can be separated. The following hypotheses were tested; (1) the abundance of adult moths is higher in margins with nectar resources than those without, (2) the diversity of moths is highest in wildflower margins and lowest in grass-only margins, (3) the abundance of grass feeding moths that also feed on nectar as adults is higher in grass margins enhanced with moth-pollinated flowers than in plain grass margins.

## Materials and methods

### Experimental design

**Fig. 1:**
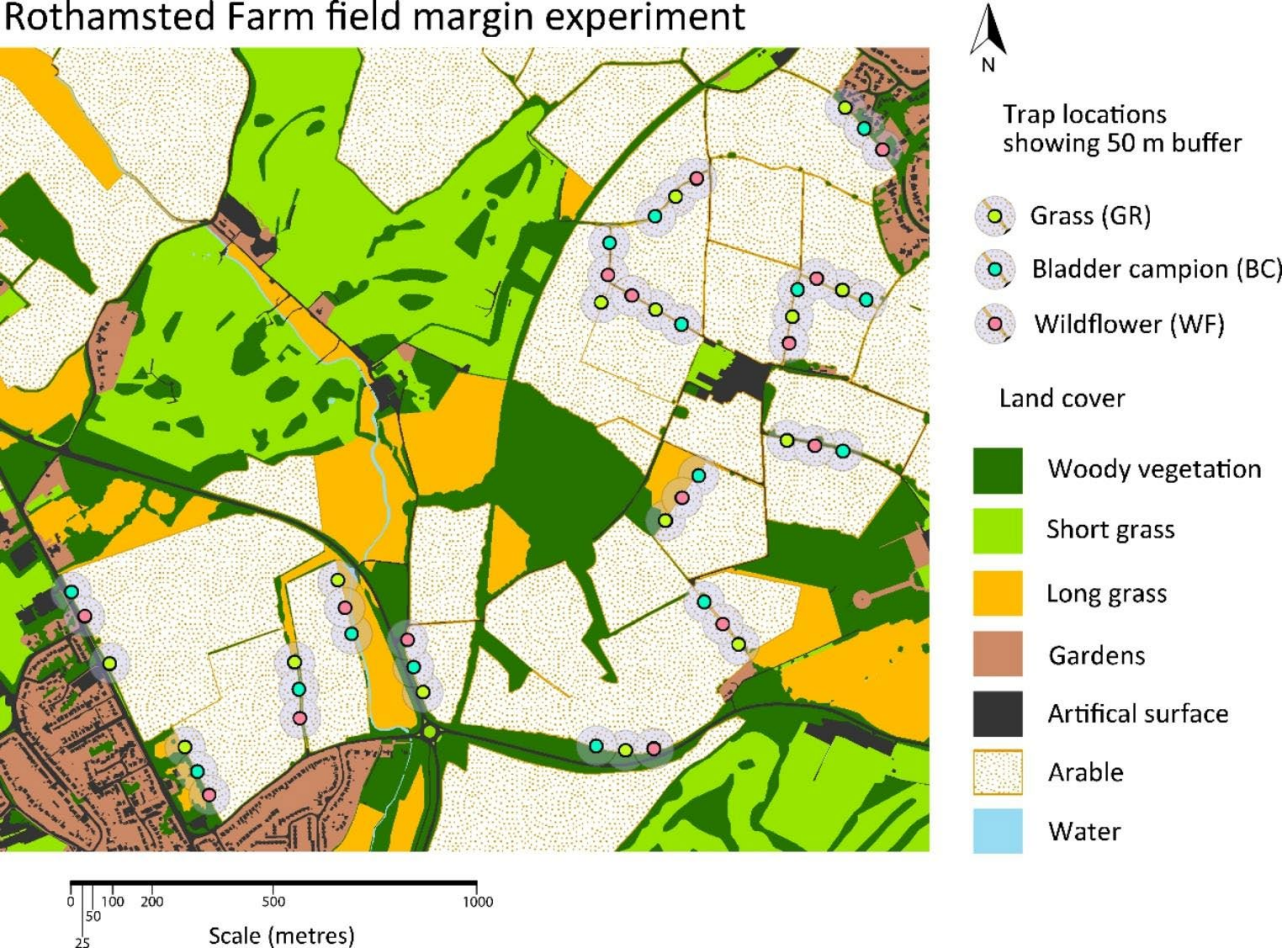
Map of randomised block design at Rothamsted Farm (51.80847, -0.38566)

A randomised block design was set up across 180 hectares on Rothamsted Farm, UK (51.808, -0.376) in April 2017. The farm is conventionally managed and is typical for the region – with a rotation of mainly wheat, oilseed rape and field beans. Fifteen field margin blocks measuring 210 × 3 m were sown at the edges of arable fields (Fig. 1). Each block was split into three plots of 70 × 3 m and one of three seed mixes (treatments) was sown into each plot within each block in a randomised order. All blocks bordered an arable field and 21 of the 45 plots also bordered a woody boundary. The three treatments were (1) ‘grass’ (GR): four species of non-competitive grasses (*Agrostis capillaris*, *Cynosurus cristatus*, *Festuca rubra* and *Phleum bertolonii*), (2) ‘Bladder Campion’ (BC): the same four grass species plus two moth-pollinated plants: *Silene vulgaris* Bladder Campion, and *Silene noctiflora* Night-flowering Catchfly, and (3) ‘wildflower’ (WF): the same four grasses plus 13 species of perennial wildflower widely used in AES margins: *Achillea millefolium* Yarrow, *Centaurea nigra* Common Knapweed, *Daucus carota* Wild Carrot, *Knautia arvensis* Field Scabious, *Leucanthemum vulgare* Oxeye Daisy, *Lotus corniculatus* Bird’s-foot Trefoil, *Malva moschata* Musk Mallow, *Primula veris* Cowslip, *Prunella vulgaris* Selfheal, *Ranunculus acris* Meadow Buttercup, *Silene dioica* Red Campion, *Trifolium pratense* Red Clover, and *Vicia cracca* Tufted Vetch. - see Supporting Information: Field margin establishment, Fig. S1 and Table S1 for details on field margin species composition, prices and sowing methods.

### Sampling procedure

Sampling of adult moths ran over two field seasons: June – September 2018 and 2019 and consisted of two techniques: light trapping and transects. Transects were used in addition to the more commonly employed light-trapping method for several reasons. Transects allowed us to observe how the moths were using the field margins (as sources of larval hostplants, nectar resources and shelter). Transects also allowed us to include species that are not attracted or only weakly attracted to light. The method also allowed us to measure the presence of moths in a more spatially precise way, as we are avoiding the potential issue of attracting moths from outside of the target habitat – a potential flaw of using only light-traps.

*Trapping*. Ultra-violet (UV) LED light-traps were deployed during eight alternate weeks each year (2018 and 2019), starting in early June and ending in mid-September, resulting in a total of 16 sample weeks. Each night from Monday - Wednesday, four blocks (i.e., 12 plots) were sampled. On the Thursday night the final three blocks were sampled, meaning that one full replicate of the experiment occurred each week. Blocks were grouped so that they were always sampled together – i.e., on Monday, blocks 1–4 were sampled and on Tuesday blocks 5–8 were sampled. UV LED traps fitted with kill jars (see Supporting Information: Trap design and Figs. S2 & S3) were placed on platforms 1 m above the ground in the centre of each plot. Traps were set automatically to switch on at sunset and off at sunrise.

*Transects*. Transect weeks alternated with trap weeks and ran from mid-June to early September. Sampling followed the same structure as for traps with four blocks sampled Monday – Wednesday and three blocks on Thursday. Within a given night, the order in which blocks were sampled was randomised. The transects began at 15 minutes past sunset and typically lasted for three to four hours. Methods were based on a modified version of the Pollard walk (Pollard and Yates, 1993). Two observers, equipped with head torches and butterfly nets walked at a slow pace (35 m per minute) along the field margin and caught and recorded every larval and adult moth that they encountered, while also recording any behavioural events observed (nectaring, mating, ovipositing and emerging from pupa); see Supporting Information (Behavioural events) for definitions. Where moth abundance was very high, only a portion of each plot was sampled – either the full plot (70 m), a half (35 m) or quarter length (17.5 m) was sampled – this was accounted for statistically in the analysis stage. In addition, larvae were sampled once using sweep-netting at the end of the season in the second year: see Supporting Information (Transect methods) for a detailed description of the transect protocols.

### Species traits

Larval feeding guilds were extracted from Waring and Townsend (2017) and Sterling and Parsons (2012). Species were defined as ‘adult nectar feeders’ if they were observed feeding on nectar at least once during the transects. Trait descriptions are shown in Tables 1 and a full list of species encountered with their corresponding traits can be found in the data provided online: Species_traits.csv. Details on moth identification and the aggregation of certain species groups are given in Supporting Information (Moth identification) and Table S2.

**Table 1:** Descriptions of subsets of species used in the analysis

Subset	Description
Woody feeder	Feeds on deciduous or coniferous trees and/or shrubs.
Grass feeder	Feeds exclusively on grasses.
Unsown forb feeder	Feeds exclusively on forbs but not on those sown in the treatments.
Sown forb feeder	Feeds either exclusively or mainly on forbs sown in the treatments.
Polyphagous	Feeds on both woody and herbaceous plants or feeds on both grasses and forbs.
Detritivores and others	Feeds on non-plant material such as fungi and bird nests. This category also includes those that feed on lichen, mosses and decaying plant material.
Adult Nectar Feeder (grass hostplant)	Species that feed exclusively on grasses as larvae and were also encountered feeding on nectar during the experiment.

### Analysis

All statistical analyses were carried out in R 4.0.3 (R Core Team, 2020). Four responses were measured: (1) adult abundance, (2) adult species richness/diversity, (3) behavioural events of adult moths and (4) larval abundance. Behavioural events were: nectaring, mating, ovipositing and emerging from pupa. For all response measures, the aim was to determine the effect of treatment (seed mix) and its potential interaction with year, while accounting for surrounding habitat. As the number of behavioural events and larvae was very low it could not be meaningfully analysed, so the results are not presented in the main text. For completeness, the methods and results for these excluded sections are presented in Supporting Information: Behavioural events and larvae.

### Abundance of adult moths

Abundance was defined as the number of moths caught per trap per night or the number of moths encountered in one 70 m transect. In cases where the full length of transect was not sampled an ‘offset’ was used in the model to account for this. Generalized Linear Mixed Models (GLMM) with a negative binomial error structure and a log-link function were fitted using the glmer.nb() function in the lme4 package (Bates et al., 2015). Fixed explanatory variables were treatment, a treatment:year interaction, woody boundary (whether or not the site was bordered by woody vegetation), woody vegetation (the area of woody vegetation with a certain radius of the site) and long grass (the area of rough grassland habitat within a certain radius of the site). The best spatial scale (25, 50 or 100 m radii) for long grass and woody vegetation were chosen by selecting the model with the lowest AICc. Woody boundary was also considered as a potential substitute for woody vegetation but was not included in the same model due to collinearity. The continuous habitat variables were square root transformed and scaled. Definitions of each surrounding habitat variable are provided in Supporting Information (Surrounding habitat variables). For transect data, the GLMMs also included temperature and temperature squared as fixed effects to account for non-linear temperature-dependant activity within a given night. Transect length was included as an offset using the ‘offset =’ argument within the glmer.nb() function. There were two partially crossed random effects: night (the night on which the sampling took place) and block (one of 15 margin blocks).

Models were run for total abundance and for each of the eight moth subsets (based on species traits – Table 1). Trap and transect data were modelled separately. The significance of each parameter was tested using a Likelihood Ratio Test (LRT). If the treatment:year interaction was found to be non-significant at the p ≥ 0.05 level then it was removed along with the year effect. Where a significant treatment effect was found (p < 0.05), pairwise post hoc tests were carried out using the emmeans() function in the emmeans package (Lenth, 2019) to determine which treatments differed from each other.

### Richness and diversity of adult moths

The species richness and diversity were calculated for each site in each year. Due to the high number of zeros and low counts of moths, diversity could not be meaningfully calculated on a nightly basis as it was for abundance, hence why the species totals are summed for each site-year. Species richness was measured as the total number of species (or aggregate taxa) recorded per site-year. Diversity was measured as the inverse of the Shannon diversity index, known as Hill number 1 or the ‘effective number of common species’. See Chao et al. (2014) for more information on Hill numbers. Data from traps and transects were analysed separately. Linear mixed effects models (LMM) were constructed using either richness or diversity as a response variable, using a Gaussian error distribution. The response was modelled as a function of treatment interacting with year plus surrounding habitat variables as described above plus a random intercept for block. The best scale for the surrounding habitat variables was found using the AICc method also described above. If the interaction between treatment and year was found to be non-significant (p ≥ 0.05) then the moth records from both years were combined, richness and diversity were recalculated for each site and the model was rerun with no year effect. Where a significant treatment effect was found (p < 0.05), pairwise post hoc tests were carried out using the emmeans() function in the emmeans package to determine which treatments differed from each other.

### Single-species responses of adult moths

To determine which species were significantly affected by treatment type, Generalized Linear Models (GLM) with negative binomial error structure were run for each species using the using the manyglm function in the mvabund package (Wang et al., 2020). All moth records were summed for each species within each plot in each year, with the two sampling methods (trap/transects) kept separate. Abundance was modelled as a function of treatment plus two surrounding habitat variables (using the same habitat variables as were used in the abundance model described above). The effect sizes for BC and WF treatment were estimated (using the GR treatment as a baseline) for each species and 95% confidence intervals around the treatment effect were calculated. Treatment effects were considered significant if their 95% confidence intervals did not include zero. Due to the large number of species tested, there was a high risk of Type 1 errors. To account for this, treatment was considered to have a consistent effect on a species if it was significant in either both sample years or using both sampling methods.

## Results

### Abundance

**Fig. 2:**
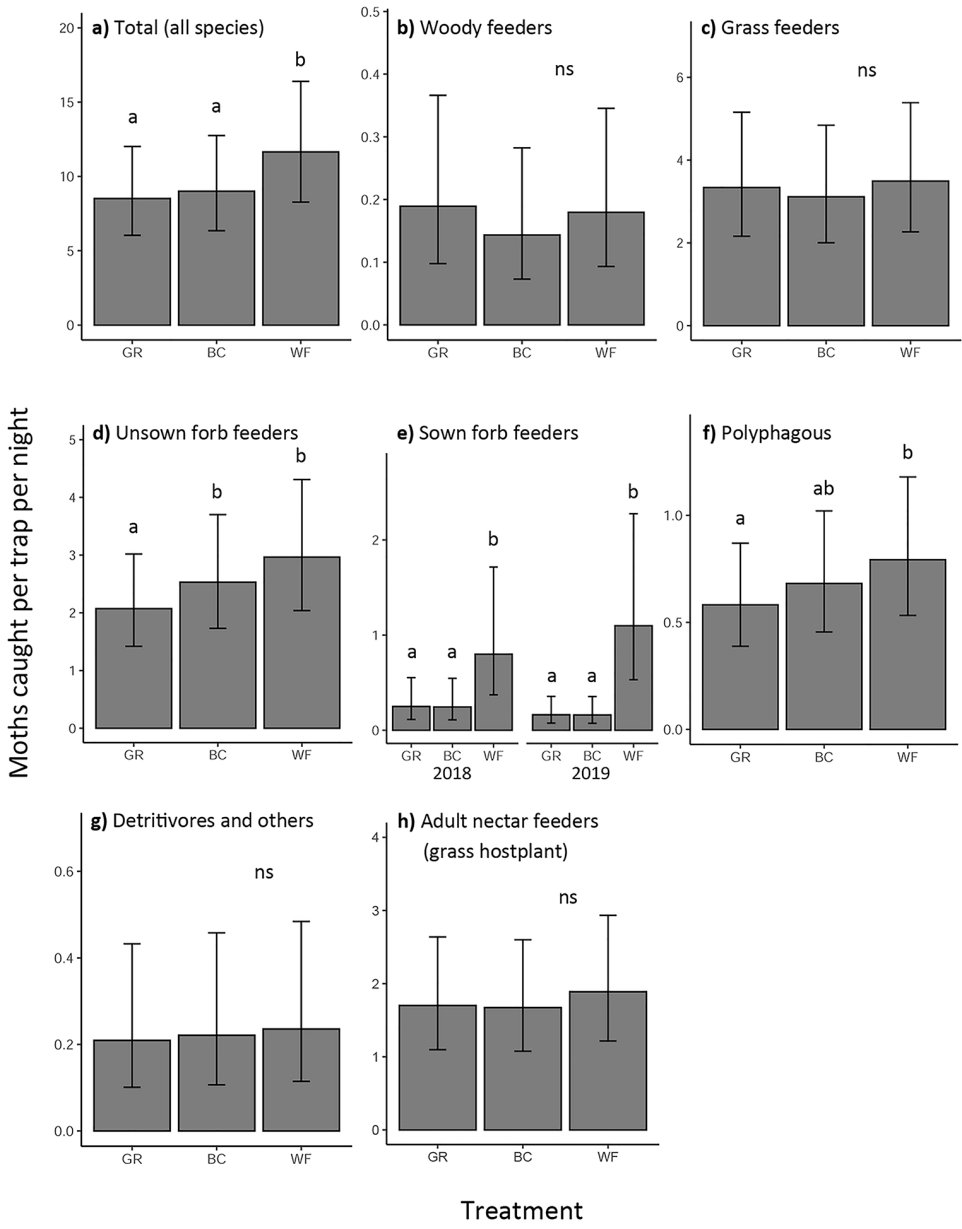
The effect of field margin treatments on moth abundance in traps. GLMM model predictions (response scale) of the expected number of moths (with 95% CIs) per trap per night with surrounding habitat variables set to their minimum with no hedgerow. Where there was a significant treatment:year interaction, the two years are plotted separately. The letters above the bars denote whether the expected counts differed between treatments according to Tukey post hoc pairwise tests at the p < 0.05 level. No significant effect is denoted by ‘ns’. GR = grass only, BC = grass plus moth-pollinated flowers, WF = grass and wildflower mix. Confidence intervals are for fixed effects only. Note that the scale on the y axes differ

*Traps*. A total of 14,769 individuals belonging to 368 taxa were caught across 711 light-trap samples (see Table S5 for a breakdown of the number of species and individuals in each group). There was a significant effect of treatment on total moth abundance (LRT, X^2^ = 48.1, p < 0.001) and on the abundance of moths belonging to feeding guilds: unsown forb feeders (LRT, X^2^ = 22.9, p < 0.001), sown forb feeders (treatment:year interaction, LRT, X^2^ = 13.9, p < 0.001) and polyphagous (LRT, X^2^ = 11.6, p = 0.004): Table S6. In all cases where treatment effect was significant, moth abundance was significantly higher in the wildflower (WF) treatment than in the grass (GR) treatment and abundance in the bladder campion (BC) treatment was typically no different from GR, apart from in unsown forb feeders where abundance in BC was the same as in WF (Fig. 2). For sown forb feeders, the effect of treatment became more pronounced in 2019. The significance tests, model parameters, post hoc test results and AICcs at different spatial scales are shown in Table S6, S7, S8 and S9 respectively.

**Fig. 3:**
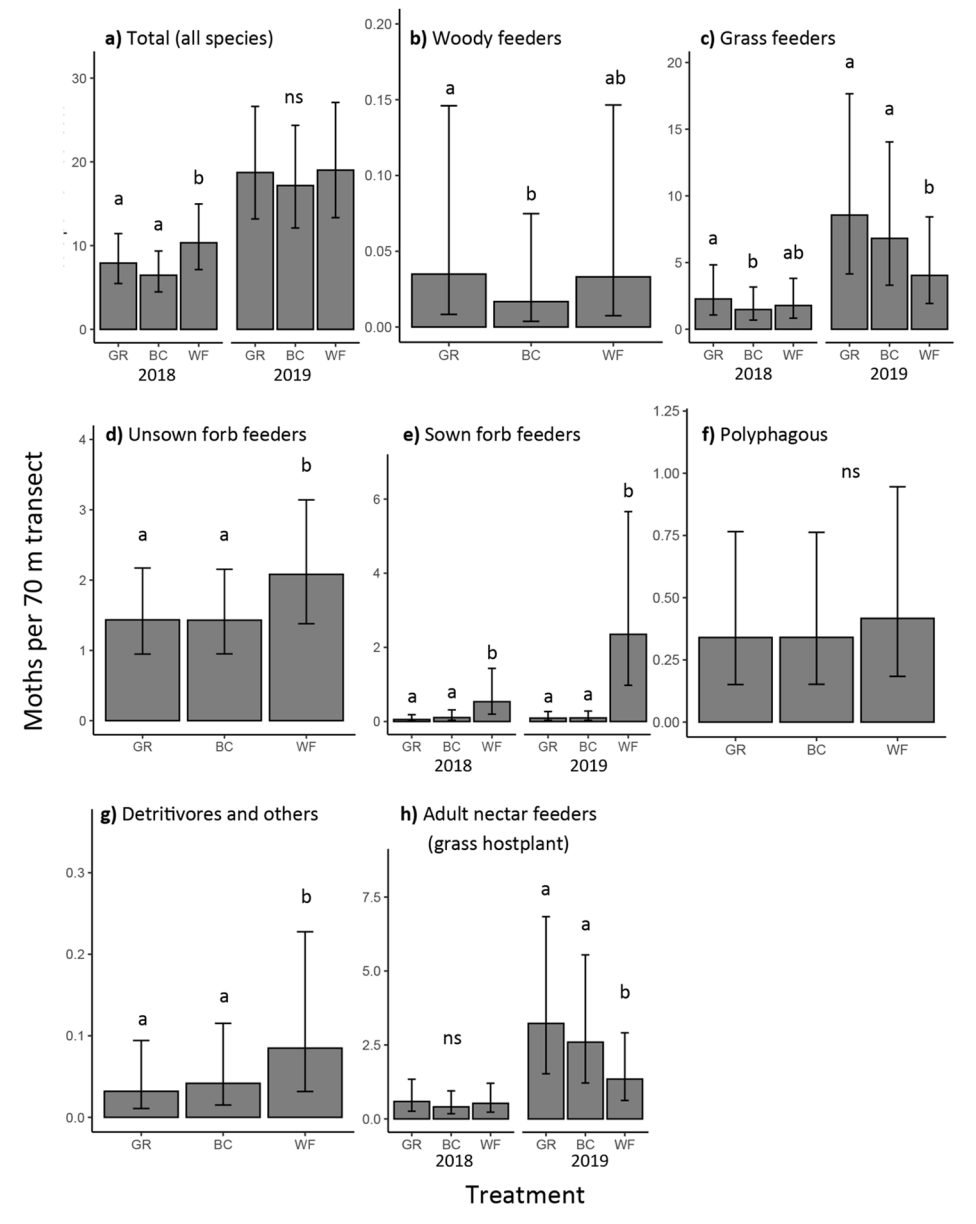
The effect of field margin treatments on moth abundance in transects. Model predictions (response scale) of the expected number of moths (with 95% CIs) for a 70 m transect on a typical night in a typical block with surrounding habitat variables set to their minimum with no hedgerow. Where there was a significant treatment:year interaction, the two years are plotted separately. The letters above the bars denote whether the expected counts differed between treatments according to Tukey post hoc pairwise tests at the p < 0.05 level. No significant effect is denoted by ‘ns’. GR = grass only, BC = grass plus moth-pollinated flowers, WF = grass and wildflower mix. Confidence intervals are for fixed effects only. Note that the scale on the y axes differ

*Transects*. A total of 5,296 adult moths belonging to 171 taxa were recorded across 516 transects. There was a significant treatment:year interaction for the total moth abundance (LRT, X^2^ = 6.3, p = 0.043) and for the abundance of grass feeders (LRT, X^2^ = 11.0, p = 0.004), sown forb feeders (LRT, X^2^ = 10.0, p = 0.007) and for species with adults found feeding on nectar (LRT, X^2^ = 12.1, p = 0.001). In years where treatment effects were significant, the effect of treatment depended on feeding guild (Fig. 3). Sown forb feeders were most abundant in the WF treatment and this effect was more pronounced in 2019. Grass feeders were most abundant in GR treatment plots and this was also more pronounced in 2019. The effect of treatment on the abundance of adult nectar feeders with grass hostplants was inconsistent between years, with no effect in 2018 and WF treatment being lower than the other two treatments in 2019. This trend was driven mainly by the grass-specialist species *Chrysoteuchia culmella* which accounted for 52% of the individuals caught in this subset in 2019. Treatment (with no year interaction) had a significant effect on unsown forb feeders (LRT, X^2^ = 11.8, p = 0.003) and detritivores/others (LRT, X^2^ = 11.7, p = 0.003) with highest counts in WF treatment. Treatment also had a significant effect on woody feeders (LRT, X^2^ = 7.3, p = 0.027) with fewer found in the BC treatment. The significance tests, model parameters, post hoc test results and AICcs at different spatial scales are shown in Table S6, S7, S8 and S9 respectively.

### Diversity

**Fig. 4:**
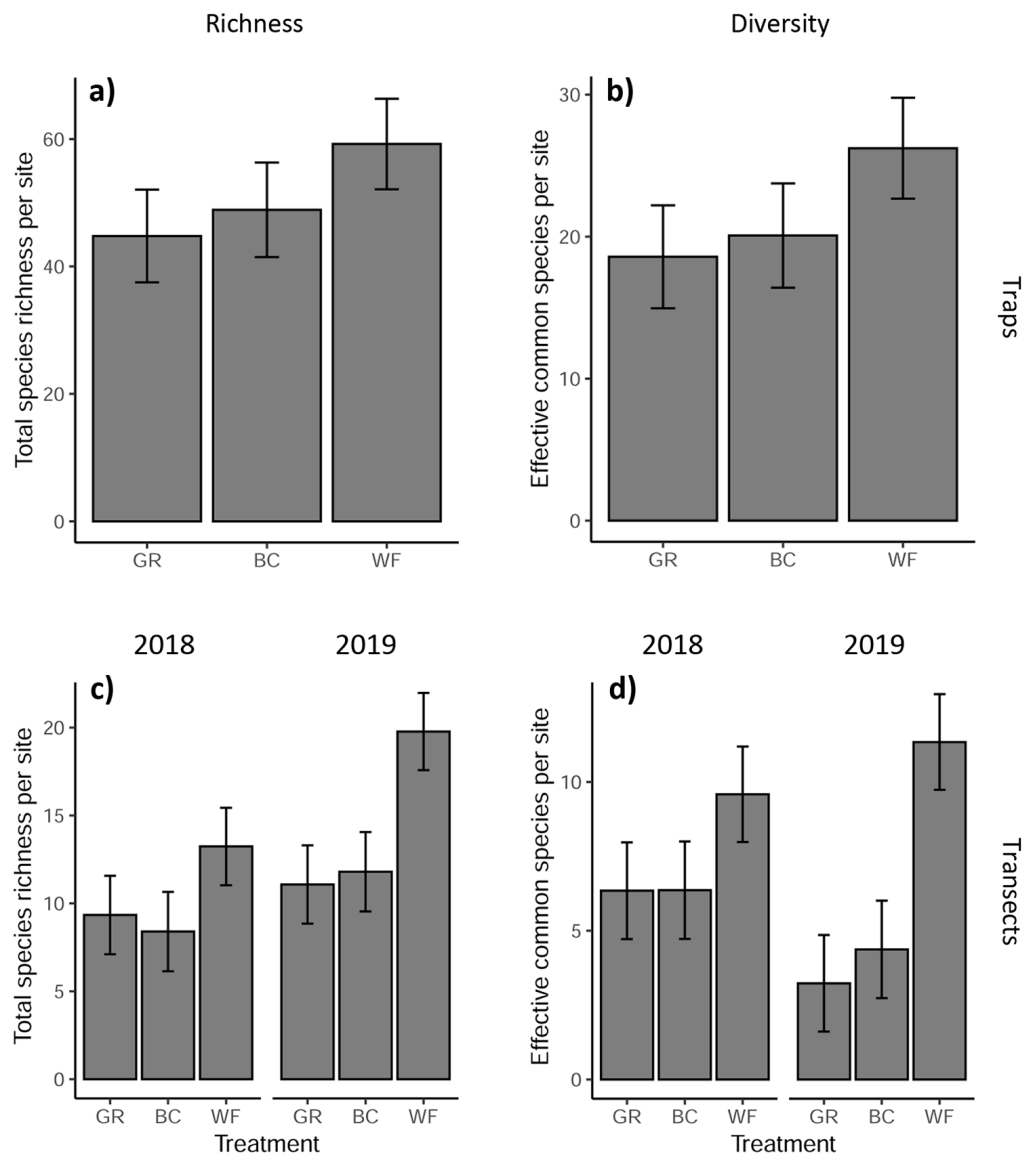
The effect of field margin treatments on moth species richness (a and c) and diversity (b and d) in traps. Diversity is measured as the exponent of the Shannon diversity index or number of ‘effective common species’. Bars show the model predictions (response scale) of the expected richness/diversity moths (with 95% CIs) for a single trap site across two years (a and b) or a 70 m transect across one year (c and d). Model predictions are for a typical block with surrounding habitat variables set to their minimum with no hedgerow. Where there was a significant treatment:year interaction, the two years are plotted separately. In all cases, Tukey post hoc pairwise tests showed that the WF treatment differed significantly from the other two treatments (p < 0.05) but there was no significant difference between the BC and GR treatments. GR = grass only, BC = grass plus moth-pollinated flowers, WF = grass and wildflower mix. Confidence intervals are for fixed effects only. Note that the scale on the y axes differ

*Traps*. After omitting nights in which a trap failed to operate, there were 219 samples of each treatment. There was no interaction between year and treatment for either species richness (LRT, X^2^ = 0.14, p = 0.93) or diversity (LRT, X^2^ = 0.68, p = 0.71) so all moth records were summed across the two years for each site. There was a significant effect of treatment on both richness (LRT, X^2^ = 29.3, p < 0.001) and diversity (LRT, X^2^ = 16.9, p < 0.001). Post hoc tests showed that for both richness and diversity, the significance of treatment was driven by a significantly higher richness/diversity in the WF treatment compared to the other two. There was no difference in richness or diversity between GR and BC treatments (Fig. 4a and b, Table S12). The ratio of WF to GR for richness and diversity was 1.3 and 1.4, respectively. The presence of a woody boundary feature and long grass habitat within a 100 m radius had significant positive effects on both richness and diversity (Table S10 and S11). The significance tests, model parameters, post hoc test results and AICcs at different spatial scales are shown in Table S10, S11, S12 and S13 respectively.

*Transects*. There was a significant﻿ interaction between treatment and year for both richness (LRT, X^2^ = 8.4, p = 0.015) and diversity (LRT, X^2^ = 12.9, p = 0.002). In each year, for both richness and diversity, the significance of treatment was driven by a significantly higher richness/diversity in the WF treatment compared to the other two. There was no difference in richness or diversity between GR and BC treatments (Fig. 4c and d, Table S12). For both richness and diversity, the difference between the WF treatment and the other two treatments became larger in the second year. For species richness, the ratio between WF and GR was 1.4 in 2018 and rose to 1.8 in 2019. For diversity, the ratio between WF and GR was 1.5 in 2018 and rose to 3.5 in 2019. As before, the presence of a woody boundary feature and long grass habitat within a 100 m radius had significant positive effects on both richness and diversity (Table S10 and S11). The significance tests, model parameters, post hoc test results and AICcs at different spatial scales are shown in Table S10, S11, S12 and S13 respectively.

### Single-species abundance models

There were 21 species for which the WF treatment was significant and 8 species for which the BC treatment was significant (Table S14). Due to the high risk of Type 1 errors, treatment was considered to have a consistent effect on a species if it was significant in either both sample years or using both sampling methods. Under this definition, there were eight species on which WF had a consistent effect: *Agriphila straminella, Bucculatrix nigricomella, Caradrina morpheus, Cochylimorpha straminea, Eucosma cana, Eucosma hohenwartiana, Idaea dimidiata* and *Pexicopia malvella*. The effect was positive in seven of these species but negative in *A. straminella.* There were no species for which the BC treatment had a consistent effect.

## Discussion

The diversity of adult moths was greatly enhanced and abundance moderately enhanced in the wildflower (WF) treatment compared to the other two treatments. Sowing diverse wildflower field margins rather than grass field margins is therefore an effective way to enhance moth abundance and diversity at the local scale. This effect was driven primarily by larval hostplant availability as the abundance of grass-feeding moths that also fed on nectar as adults was no higher in grass plots enriched with moth-pollinated flowers (BC) than in plain grass (GR) plots. For moths that did not specialise on forbs as a hostplant, abundance in the WF treatment was typically no higher in the WF treatment compared to the other two treatments, suggesting that the nectar resources provided by the WF treatment did not strongly affect the abundance of moths. The strongest effects of treatment were found for species whose larval hostplants were sown in the WF treatment. Our findings align with Alanen et al. (2011) who found that larval hostplant was more important in determining the species richness of diurnal moths than nectar sources.

Several previous studies have demonstrated that AES field margins enhance moth abundance and diversity when compared to a control (Alison et al., 2016, Merckx et al., 2012), but there are few studies that investigate the effect of different kinds of AES field margin. Studies on butterflies show an increase in abundance and diversity of butterflies in wildflower strips compared to grass or natural regeneration (Aviron et al., 2006, Pywell et al., 2007). In contrast to the findings in our experiment, several studies on butterflies have shown that nectar is a more important predictor than the presence of larval hostplants (Clausen et al., 2001, Feber et al., 1996). Our experiment agrees with previous work on other insect taxa in that wildflower field margins typically support a higher abundance and diversity of invertebrate species when compared to grass-only margins.

By modelling the response to treatment for each species separately, it was possible to determine which species benefitted most from the treatments (see Table S14). There were eight species that had a consistent response to WF treatment. Five of these species are specialists of hostplants sown in the WF treatment: *Bucculatrix nigricomella, Cochylimorpha straminea, Eucosma cana, Eucosma hohenwartiana, Pexicopia malvella*. Of these, *C. straminea E. cana* and *E. hohenwartiana* all feed on *Centaurea nigra* Knapweed which was a sown species in the WF treatment. *Bucculatrix nigricomella* feeds on *Leucanthemum vulgare* Oxeye Daisy which was also highly abundant in the WF treatment. *Pexicopia malvella* feeds on *Alcea sp.* Hollyhock and related species and it was likely feeding on *Malva moschata* Musk Mallow which was a sown forb abundant in the WF treatment. *Agriphila straminella* is a grass specialist and had lower abundance in the WF treatment, likely due to the lower density of grass in this treatment type. In contrast, there were no species on which the BC treatment had a consistent effect.

In ecological studies, moths are typically only sampled in their adult stage, but presence of adults in a habitat does not necessarily imply successful breeding. It has been suggested that annually ploughed nectar-rich field margins may act as a population sink, drawing in adult insects but inflicting high overwintering mortality (Ganser et al., 2019). In our study, the number of larvae counted was too few for meaningful analysis (77 larvae were counted during transects across the two years and 40 during sweep-net surveys: see Supplementary Information: Behavioural events and larvae), so the effectiveness of these sown field margins as breeding habitat cannot be confirmed definitively. However, the effectiveness of perennial field margins as overwintering sites has been demonstrated for numerous insect taxa (Ganser et al., 2019, Pfiffner and Luka, 2000, Schaffers et al., 2012), but comparable studies on Lepidoptera appear largely absent. Despite lack of direct evidence, our data showed that treatment effects became more prominent in the second year of the study, suggesting that populations of moths specialising on the sown species had established. In line with previous studies, this trend is expected to continue in subsequent years as more species colonise the new habitat (Alanen et al., 2011, Korpela et al., 2013). Furthermore, although recorded only infrequently, we did encounter all stages of the moth life cycle occurring within the margins: ovipositing, larvae, emerging from pupa, and mating. Empty pupal cases of burnet moths were also frequent in the WF treatment, but these were not quantified. The use of emergence traps would be useful in further studies to confirm that moths are indeed overwintering in perennial field margin habitats.

Field studies on moths typically employ light-traps as a sampling method, only rarely using transects (e.g., Birkinshaw and Thomas, 1999). In our study, the results from these two methods both corroborated that species richness and diversity was higher in WF treatment compared to the other treatments. They also supported our finding that the abundance of sown forb feeders was substantially higher in WF treatment than in the other two treatments and that this was more pronounced in 2019. We found that trapping recorded over twice as many species as transects (see Table S5) and therefore represents a larger proportion of the moth community. Our findings suggest that moth-trapping offers an effective and more time-efficient method of sampling if the researchers are interested only in presence and abundance of moths rather than observing how they are using the habitat.

This experiment showed that farm scale moth diversity was greatly enhanced by sowing diverse grass and wildflower field margins rather than plain grass margins in arable fields. Local abundance was also enhanced, but this effect was less clear: ranging from no effect, to a 1.4-fold increase when comparing wildflower margins to grass margins. The benefit of wildflower margins for moths was driven primarily by their role as a larval hostplant; their role as a source of nectar for adult moths appears of secondary importance. We found that *Centaurea nigra* Knapweed was especially beneficial to moths with a measurable effect on three species (listed above). We therefore recommend that this plant be included in seed mixes when sowing wildflower field margins.

The value of wildflower field margins tends to increase with age (Alanen et al., 2011) as more species colonise the new habitat over time. In this experiment, the effects of the wildflower margins were more pronounced in the second year, highlighting the importance of maintaining long-term semi-natural habitats on farmland. Sown field margin strips are an important tool in mitigating biodiversity loss in arable farmland and connecting existing areas of semi-natural grassland. Here it is shown that, for moths, the small amount of space allotted to sown field margins can be used more effectively to enhance local abundance and diversity by sowing a diverse range of wildflowers and grasses rather than plain grass. However, the gains in abundance are modest and it is likely that the preservation and creation of larger areas of habitat is needed to halt the decline in the abundance of moths.

## Electronic supplementary material

Below is the link to the electronic supplementary material.

Supplementary Material 1

## Data Availability

The dataset and R code necessary to replicate the analysis will be published with a DOI on the Rothamsted Research Repository (https://repository.rothamsted.ac.uk/) under a Creative Commons Attribution 4.0 Licence.
