# Supplementary material for: Floral enhancement of arable field margins increases moth abundance and diversity
Source: J Insect Conserv. 2023 Mar 23;27(3):455–65. doi: 10.1007/s10841-023-00469-9 (PMC10205847; doi:10.1007/s10841-023-00469-9)
Supplement: Supplementary file 1 — Supplementary Material 1 [file 10841_2023_469_MOESM1_ESM.docx]

Supporting information - Floral enhancement of arable field margins increases moth abundance and diversity

## Field margin establishment

Mid-April 2017: The 210 x 3 m margins were marked out and a herbicide (glyphosate) was applied to the plots to kill off any crops or pre-existing vegetation.

Late April 2017: The seeds were sown using a combination drill that cultivates in front of the drilling harrows. The drilling harrows lift right out of the ground so the seed is sprinkled on top, similar to being broadcast. Another set of harrows then followed behind the drilling harrows and lightly raked the soil, covering the seed. The soil was then rolled to consolidate the seed bed.

Late June 2017: Margins were cut close to ground level to suppress the annual weeds.

Mid-August 2017: Margins were cut again close to the ground to suppress annual weeds. Sown perennials now starting to establish.

May – September 2018: Field season 1. Some margins suffered lodging (vegetation falling over).

Late September 2018: Margins were cut and vegetation removed.

Mid-May 2019: The margins that suffered lodging last season (4 out of 15 margins) were cut to reduce the likelihood of lodging this year.

April – September 2019: Field season 2.


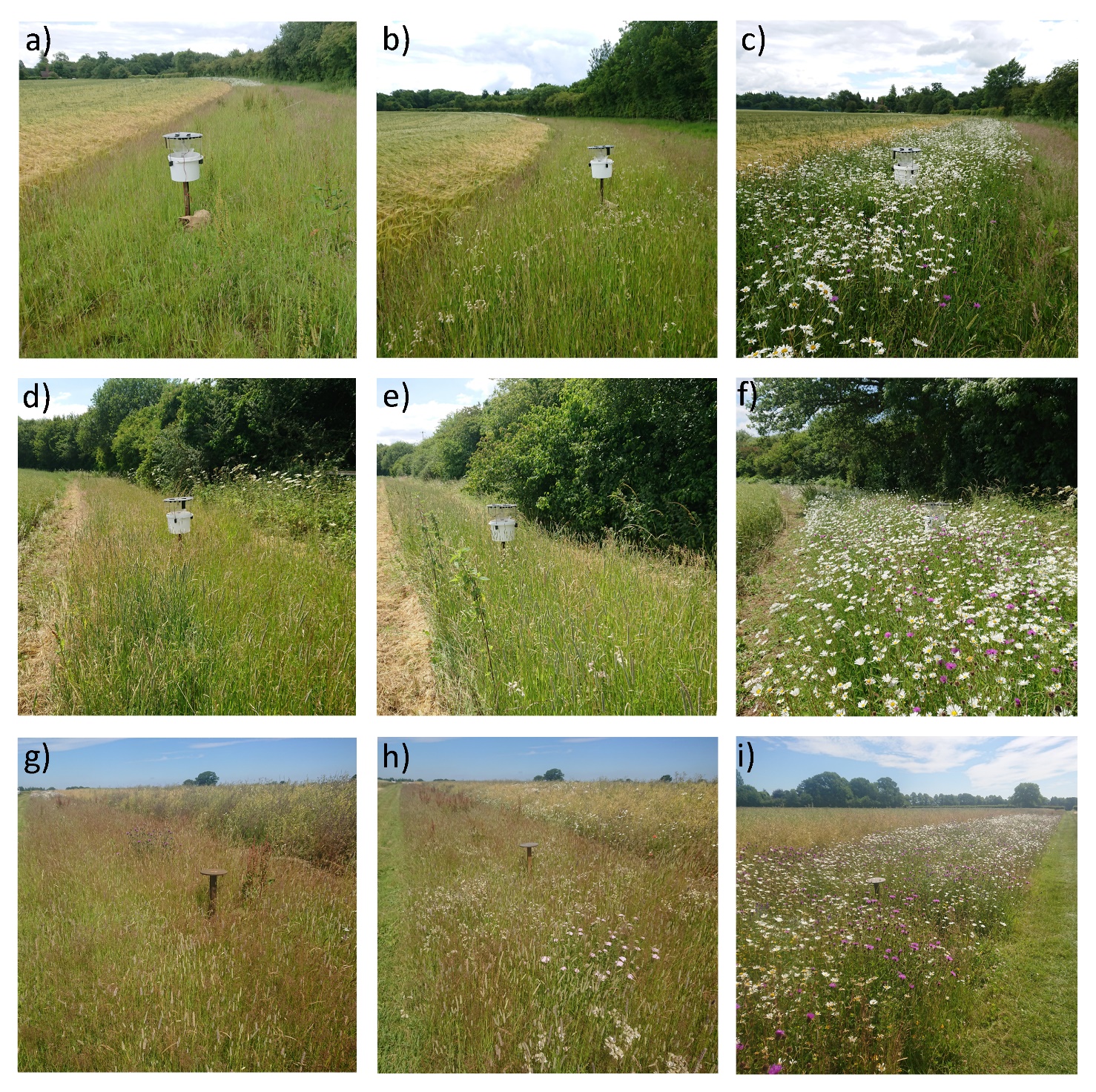


Fig. S1. Photos of field margin blocks at Rothamsted Farm, showing the three treatments. Grass (GR): a, d, g, Bladder campion (BC): b, e, h, and Wildflower (WF): c, f, i. Photos taken in 2019 on June 20th (a – c), July 3rd (d – f) and July 5th (g – i). Traps on a 1 m platform are shown in a – f and only the platforms are shown in g – i.

Table S1. Species composition, sowing rates and price per hectare of the three seed mixes (treatments) used in the experiment. All seed mixes were sown at a rate of 10 kg/ha.

| Seed mix | Price (£/ha) | Common name | Scientific name | Percentage composition |
| --- | --- | --- | --- | --- |
| Grass (GR) | £104 | Common bent | *Agrostis capillaris* | 10% |
|  |  | Crested dogstail | *Cynosurus cristatus* | 50% |
|  |  | Slender-creeping red-fescue | *Festuca rubra* | 35% |
|  |  | Smaller cat's-tail | *Phleum bertolonii* | 5% |
| Bladder campion (BC) | £191 | Common bent | *Agrostis capillaris* | 9% |
|  |  | Crested dogstail | *Cynosurus cristatus* | 46% |
|  |  | Slender-creeping red-fescue | *Festuca rubra* | 32% |
|  |  | Smaller cat's-tail | *Phleum bertolonii* | 5% |
|  |  | Night-flowering catchfly | *Silene noctiflora* | 6% |
|  |  | Bladder campion | *Silene vulgaris* | 2% |
| Wildflower (WF) | £360 | Common bent | *Agrostis capillaris* | 8% |
|  |  | Crested dogstail | *Cynosurus cristatus* | 40% |
|  |  | Slender-creeping red-fescue | *Festuca rubra* | 28% |
|  |  | Smaller cat's-tail | *Phleum bertolonii* | 4% |
|  |  | Yarrow | *Achillea millefolium* | 1.2% |
|  |  | Common knapweed | *Centaurea nigra* | 3% |
|  |  | Wild carrot | *Daucus carota* | 1% |
|  |  | Field scabious | *Knautia arvensis* | 0.6% |
|  |  | Oxeye daisy | *Leucanthemum vulgare* | 1.6% |
|  |  | Birdsfoot trefoil | *Lotus corniculatus* | 2% |
|  |  | Musk mallow | *Malva moschata* | 0.8% |
|  |  | Cowslip | *Primula veris* | 0.4% |
|  |  | Selfheal | *Prunella vulgaris* | 3% |
|  |  | Meadow buttercup | *Ranunculus acris* | 3.2% |
|  |  | Red campion | *Silene dioica* | 2% |
|  |  | Wild red clover | *Trifolium pratense* | 0.2% |
|  |  | Tufted vetch | *Vicia cracca* | 1% |

## Trap design

The ‘light bulb’ unit was made from a strip of ultra-violet (UV) LEDs (x 30 LED units, chip type 3528 SMD, 12 volts) wrapped around a plastic cylinder inserted into a clear polystyrene 30 ml casing (Fig. S4). The bulb unit had an output power of less than 3 W and was powered by a lead-acid 12-volt battery. The LEDs had a narrow spectral emittance from 395 – 405 nm with a peak at 400 nm, effectively on the edge of the UV and visible spectrum, and hence appearing to the human eye as violet. The light bulb was connected to an automatic clock timer enclosed in a waterproof plastic clip-lock box. The clock timer was programmed manually to switch on at sunset and off at sunrise. The light bulb was suspended above a funnel (upper and lower diameter: 220 and 70 mm respectively) which led into a kill-jar: a 1-litre Kilner jar lined with plaster (gypsum) and infused each night with tetrachloroethylene as is used by the Rothamsted Insect Survey. Fig. S3 shows the full trap.


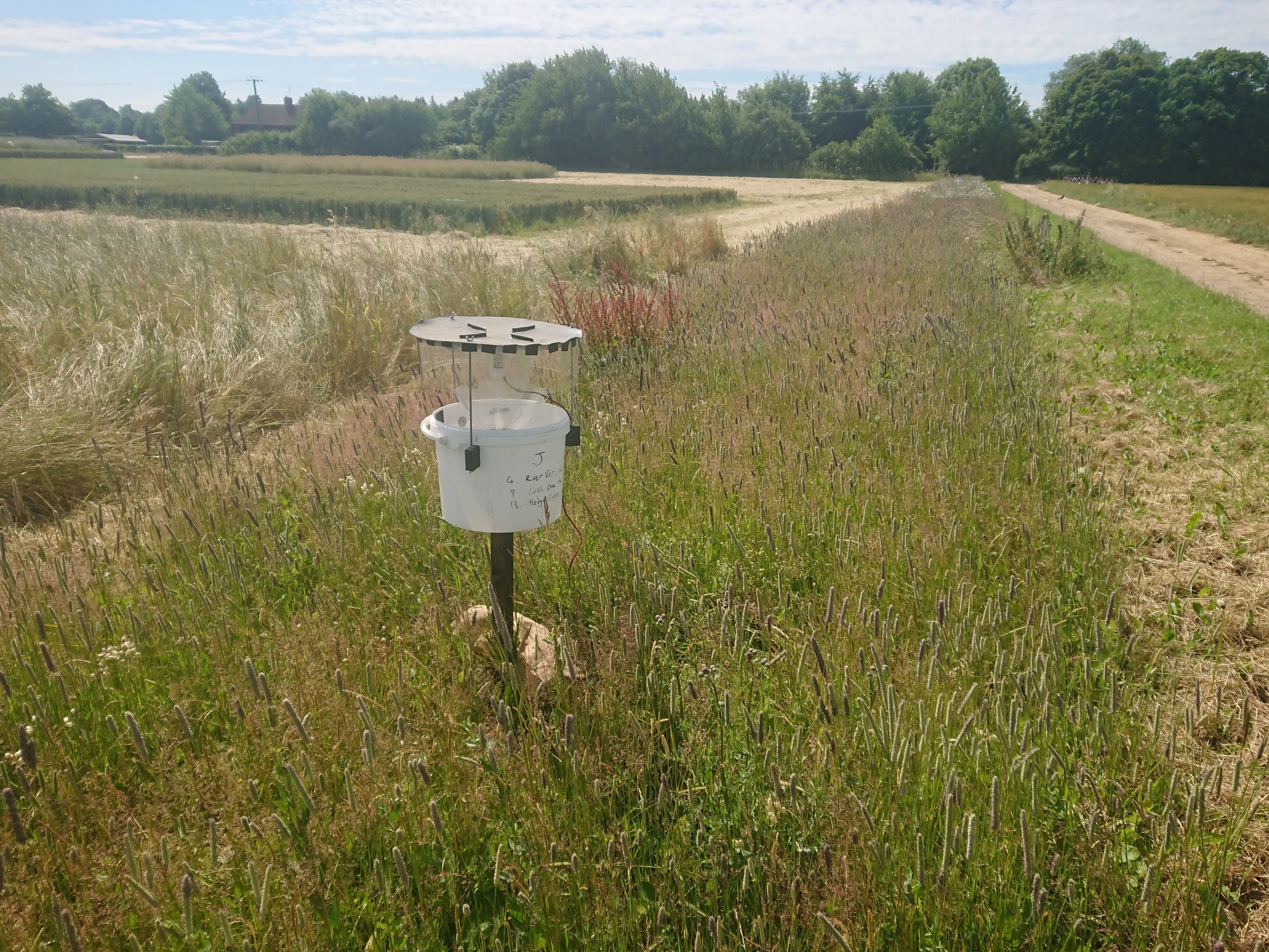


Fig. S2. A moth trap used in the study. The funnel leads to a killing jar which is inside the bucket. The battery and clock timer are inside the waterproof hessian bag on the ground.


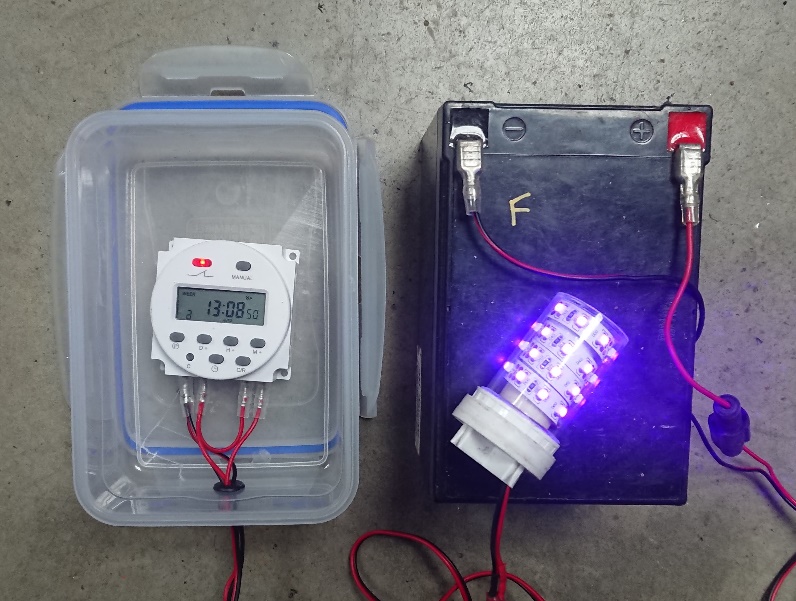


Fig. S3. The electronic clock, light and battery setup for the UV-LED moth-trap.

## Behavioural events

Behavioural events we recorded were nectaring, mating, ovipositing and emerging from pupa. We defined these as such:

Nectaring: Any moth seen at a flower with its proboscis extended and the proboscis making contact with the nectar-producing part of the flower.

Mating: Any pair of moths in copulation.

Ovipositing: A moth observed laying eggs. Oviposition was only confirmed if the eggs could be seen.

Emerging from pupa: Any moth found that was freshly emerged and in the process of expanding its wings. Or, in the case of above-ground pupae, any moth seen emerging.

## Transect methods

Two observers were each equipped with head torches (Black Diamond, Cosmo), butterfly nets and a bag of plastic sample pots and two empty bags. Head torches were used with white light setting at the default level brightness. Although using red light mode would be less disruptive, experience showed that this was impractical due to low visibility. We found that, despite the possibility of moths being attracted to the light, moths found engaging in behaviour such as nectaring or mating did not show any obvious signs of disturbance when illuminated. Transects began at 15 minutes past sunset. The observers began at one end of the margin block, noting the time and the temperature. The direction in which the block was sampled was alternated weekly. One observer walked at 1 m in from the crop edge, in the sown vegetation, the other observer walked on the edge between the sown strip and the incidentally occurring vegetation on the side further from the crop. The observers walked at a very slow pace (35 m per minute) and scanned a radius of 1.5 m around them, excluding anything 0.5 m above head height in a manner similar to a Pollard walk (Pollard and Yates, 1993). The surveyed area thus included the 3 m width of the sown strip, plus 0.5 m into the crop and 1.5 m into the incidentally growing vegetation.

When a moth (adult or larva) was encountered, the individual was caught and placed in a sample pot. Both observers stopped walking and the observer without the moth turned off their head torch. If the moth could be identified in-field, its identity was recorded and the sample pot was placed into bag no. 1. If it could not be identified, or if it was a larva, a code was written both in the notebook and on the sample pot and it was put in bag no. 2. Any behaviours that were witnessed (nectaring, mating, ovipositing or emerging from pupa) were recorded. At the end of a block, all moths in bag no. 1 were released but moths in bag no. 2 were kept and the pots containing adults were put in a refrigerator overnight to be identified in the morning. Larvae were reared to adulthood indoors at ambient temperature and provided with the hostplant that they were found on.

On nights where moth abundance was low, the entire length of each 70 m of each plot was sampled. Where moth abundance was moderate to high, the plots were subsampled by only sampling the first 35 m of each plot. On several occasions in July and August 2019, the abundance was so high that subsampling of 17.5 m sections was required so that all plots could be sampled within the night. The discrepancies in distance covered was later accounted for statistically by using an offset so the response became moths per unit distance surveyed. Subsampling levels were always applied equally to every plot within a block. A sample night typically took 3 to 4 hours.

## Moth identification

All moths were identified to species level where possible and dissected if necessary. Several species groups were aggregated. These were groups that were either too difficult to identify even by dissection, were too numerous to dissect, or both. These nine aggregate groups are only a small proportion of the roughly 400 species recording during the experiment, so using these aggregate taxa is unlikely to affect species richness/diversity indices in a meaningful way. Species aggregates are shown in Table S2.

Table S2. The nine groups of species that were combined into aggregate taxa for analysis purposes.

| Aggregate name | Macro/micro moth | Species included in aggregate |
| --- | --- | --- |
| *Cnephasia* agg. | Micro | All species in the *Cnephasia* genus excluding C*. longana* |
| *Ectodemia* sp. | Micro | All species in *Ectodemia* genus |
| *Mesapamea* agg. | Macro | *Mesapamea secalis* and *M. didyma* |
| *Monopis* agg. | Micro | *Monopis laevigella* and *M. weaverella* |
| *Oegoconia sp.* | Micro | All species in *Oegoconia* genus |
| *Parornix* sp. | Micro | All species in *Parornix* genus |
| *Phyllonorycter sp.* | Micro | All species in P*hyllonorycter* genus |
| *Scoparia* agg. | Micro | *Scoparia ambigulais* and *S. pyralella* |
| *Stigmella* sp. | Micro | All species in *Stigmella* genus |

## Behavioural events and larvae

### Methods

*Behaviour of adult moths*. The occurrence of the four behavioural events (nectaring, mating, ovipositing and emerging from pupa) were summed within each of the 45 plots across the two years. Only events occurring inside the sown strip were considered. Generalised Linear Mixed Models (GLMMs) with negative binomial error structures were constructed for each of the four behaviours, with the total number of events observed per plot as the response variable. The glmer.nb() function in the lme4 package was used. Treatment was always included as a fixed effect and the best spatial scale (25, 50 or 100 m) for the long grass and woody vegetation (or woody boundary) variables were determined by running models with each of the 12 possible combinations and selecting the model with the lowest AICc. Block was included as a random intercept. The significance of the parameters was determined using Likelihood Ratio Tests. All parameters were left in the final model even if not significant at the p < 0.05 level.

To determine the relative visitation rates of flowers/fruits as a nectar/sugar source, all observations of nectaring events were summed up for each plant species across the entire experiment, including observations that occurred outside of the sown strips.

*Larval abundance*. In addition to larvae encountered during the transects, a sweep-net survey for larvae was carried out in September 2019. This was done at night, during the final transect of the season. Two people swept the vegetation with sweep-nets while walking, taking 50 sweeps per person per treatment plot. In both methods (transects and sweep-netting), all larval counts were summed up for each of the 45 plots across the two years. Abundance from the two methods was modelled separately. Only larvae found within the sown strips were considered. For each method, a GLMM with a negative binomial error structure was constructed with the same modelling procedure as described above.

### Results

*Nectaring*

Within the sown margins, a total of 62 nectaring events were observed involving 27 species of moth and 11 species of plants (see online data Behavioural_events.csv). There was a significant effect of treatment on the number of nectaring events observed (*X^2^* = 20.7, p < 0.001) and a significant negative effect of the amount of long grass habitat within 50 m of the site (*X^2^* = 6.94, p = 0.008). Post-hoc tests showed that there were equivalent numbers of nectaring events in the WF and BC treatments but fewer in the GR treatment (Fig. S5).


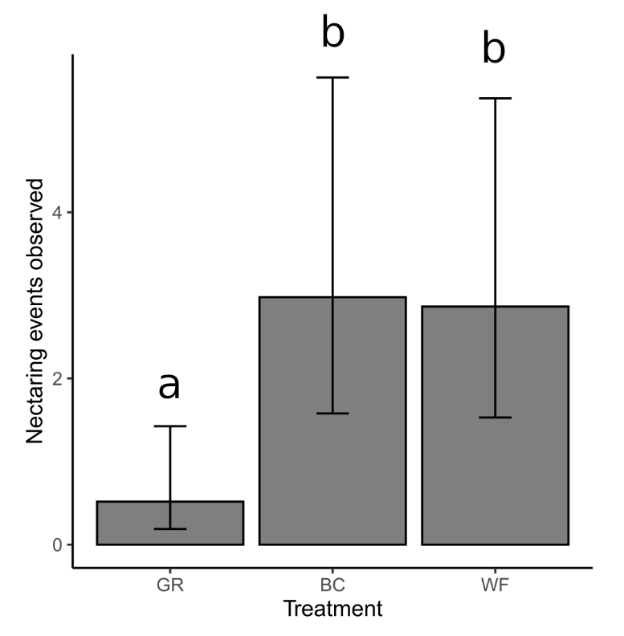


Fig. S4. Effect of field margin treatment on nectaring events observed. Model predictions (response scale) of the total expected number of events for a single site (with 95% CIs) in a typical block with surrounding habitat variables set to their minimum with no hedgerow. A post-hoc Tukey test was carried out on pairwise comparisons of treatment levels. The letters above the bars denote whether the expected counts differed between treatments. GR = grass only, BC = grass plus moth-pollinated flowers, WF = grass and wildflower mix. Confidence intervals are for fixed effects only.

*Mating and other observations*. A total of 36 mating events (i.e. 72 moths) were recorded within the sown strips. 67% of these were *Xestia xanthographa* (Square-spot Rustic). The other species were two pairs of *Korscheltellus lupulina* (Common Swift), two pairs of *Triodia sylvina* (Orange Swift) and one pair each of *Agapeta hamana, A. zoegana*, *Eucosma cana*, *Hepialus humuli* (Ghost Moth), *Mesoligia furunculi* (Cloaked Minor)*, Mythimna impura* (Smoky Wainscot), *Pterophorus pentadactyla* (White Plume) and *Zygaena filipendulae* (Six-spot Burnet). There was no effect of treatment (*X^2^* = 0.69, p = 0.71), woody boundary (*X^2^* = 2.44, p = 0.12) or long grass within a 100 m radius (*X^2^* = 0.62, p = 0.80). Oviposition was observed only twice, both in 2018. *Spilosoma lubricipeda* (White Ermine) was observed ovipositing on oxeye daisy in the WF treatment and *Sideridis rivularis* (The Campion) was observed ovipositing in night-flowering catchfly in the BC treatment. One *Apamea monoglypha* (Dark Arches) was also found freshly emerged from its pupa and expanding its wings in the GR treatment.

*Larval abundance*. A total of 77 larvae belonging to 9 identified species were recorded during transects in the sown strips. 95% of larvae found were either known grass feeders or were found feeding on grass. Only two larvae (both *Noctua fimbriata,* Broad-bordered Yellow Underwing) were found feeding on a sown wildflower, this being wild carrot in both cases. In the sweep-net samples in September 2019, a total of 40 larvae belonging to at least four species (but only one identified species) were caught (Table S3). For larvae encountered during transects, there was no significant effect of treatment (*X^2^* = 5.64, p = 0.060) or woody vegetation within a 25 m radius (*X^2^* = 4.13, P = 0.27). There was a significant positive effect of the amount of long grass within a 25 m radius (*X^2^* = 4.13, p = 0.04). See Table S4 for effect sizes. In the sweep-net samples, there was a significant effect of treatment (*X^2^* = 12.4, P = 0.002, Fig. S7), with a post-hoc tests showing that larval abundance was lower in the WF treatment than in the other two treatments. For sweep net surveys, there was no effect of woody boundary (*X^2^* = 1.64, p = 0.20) or long grass within a 100 m radius (*X^2^* = 0.62, p = 0.43).


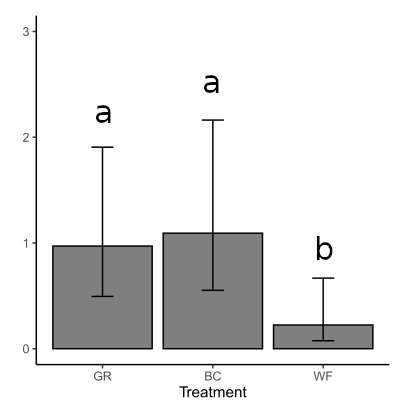


Fig S5. The number of larvae caught per 50 sweeps in sweep net samples in September 2019.

Table S3. Summary of the species recorded in larval form during the entire 2-year experiment within sown treatments.

| Common name |  | Number of larvae found visually during transects | Number of larvae caught in sweep net samples |
| --- | --- | --- | --- |
| Broad-bordered Yellow Underwing | *Noctua fimbriata* | 2 | 0 |
| Clay | *Mythimna ferrago* | 4 | 0 |
| Common Wainscot | *Mythimna pallens* | 4 | 0 |
| Deep Brown Dart | *Aporophyla lutulenta* | 1 | 0 |
| Drinker | *Euthrix potatoria* | 1 | 0 |
| Geometrid sp. |  | 4 | 0 |
| Lesser Yellow Underwing | *Noctua comes* | 1 | 0 |
| Noctuid sp. |  | 40 | 22 |
| Other sp. |  | 6 | 2 |
| Setaceous Hebrew Character | *Xestia c-nigrum* | 1 | 0 |
| Smoky Wainscot | *Mythimna impura* | 12 | 0 |
| Square Spot Rustic | *Xestia xanthographa* | 1 | 0 |
| Mother Shipton | *Callistege mi* | 0 | 16 |
| **TOTAL** |  | **77** | **40** |

Table S4. Model summaries for larvae and behavioural events. Significant effects (p < 0.05) are in bold.

| Method | Response | Parameter | Estimate | Std. error | Z | p-value |
| --- | --- | --- | --- | --- | --- | --- |
| Larvae counts | Total larval abundance | Intercept | -0.074 | 0.342 | -0.215 | 0.830 |
|  |  | Treatment BC | 0.373 | 0.266 | 1.398 | 0.162 |
|  |  | Treatment WF | -0.329 | 0.317 | -1.038 | 0.299 |
|  |  | Woody 25m | 0.265 | 0.236 | 1.121 | 0.262 |
|  |  | Long grass 25m | 0.558 | 0.266 | 2.095 | 0.036 |
| Sweep net samples | Total larval abundance | Intercept | -0.040 | 0.323 | -0.124 | 0.901 |
|  |  | Treatment BC | -0.118 | 0.335 | -0.353 | 0.724 |
|  |  | Treatment WF | -1.583 | 0.552 | -2.865 | 0.004 |
|  |  | Boundary | 0.480 | 0.365 | 1.314 | 0.189 |
|  |  | Long grass 100m | -0.156 | 0.200 | -0.779 | 0.436 |
| Behaviour | Nectaring events | Intercept | -1.148 | 0.500 | -2.296 | 0.022 |
|  |  | **Treatment BC** | **1.752** | **0.493** | **3.551** | **<0.001** |
|  |  | **Treatment WF** | **1.713** | **0.495** | **3.464** | **<0.001** |
|  |  | Boundary | -0.665 | 0.409 | -1.625 | 0.104 |
|  |  | **Long grass 50m** | **-0.595** | **0.225** | **-2.65** | **0.008** |
|  | Mating events | Intercept | -0.064 | 0.341 | -0.189 | 0.850 |
|  |  | Treatment BC | 0.157 | 0.391 | 0.401 | 0.689 |
|  |  | Treatment WF | -0.186 | 0.426 | -0.438 | 0.661 |
|  |  | Boundary | -0.695 | 0.433 | -1.606 | 0.108 |
|  |  | Long grass 100m | -0.048 | 0.192 | -0.251 | 0.802 |

## Surrounding habitat variables

Around the centre of each plot, buffers of radii 25, 50 and 100 m were drawn using ArcMap (version 10.4). The proportions of two habitat types (1) woody vegetation and (2) long grass were calculated for each buffer size at each plot. These two habitats were chosen as they are known to be the most important determinants of moth abundance and diversity at the farm scale (Woiwod and Gould, 2008). Long grass included rough grassland and any other semi-natural low-growing vegetation (see map, Fig. S1). Woody vegetation included hedgerows, woodlands and solitary trees. In addition to the continuous variables, a two-level factor variable was also used to describe whether each plot was next to a linear woody boundary feature or not. This was included as moths are known to use linear woody boundary features as dispersion corridors (Coulthard *et al.*, 2016). Woody boundary features could be either hedgerows, the edges of dense stands of trees or the edge of woodlands.

Table S5. Summary statistics of moths recorded during the entire study. Showing the total number of individuals caught and the number of species caught in each subset. Percentage in brackets shows the percentage that each subset represents of the whole.

|  |  | Total individuals caught | Total species caught |
| --- | --- | --- | --- |
| Traps | All moths | 14769 | 368 |
|  |  |  |  |
|  | Woody | 1134 (7.7%) | 121 (33%) |
|  | Grass | 4834 (33%) | 47 (13%) |
|  | Unsown forb | 3709 (25%) | 105 (29%) |
|  | Sown forb | 1070 (7.2%) | 31 (8.4%) |
|  | Polyphagous | 1555 (11%) | 20 (5.4%) |
|  | Detritivores and others | 2366 (16%) | 41 (11%) |
|  | Adult nectar feeders (grass hostplant) | 2957 (20%) | 10 (2.7%) |
|  |  |  |  |
| Transects | All moths | 5296 | 171 |
|  |  |  |  |
|  | Woody | 81 (1.5%) | 27 (16%) |
|  | Grass | 2858 (54%) | 31 (18%) |
|  | Unsown forb | 725 (14%) | 56 (33%) |
|  | Sown forb | 376 (7.1%) | 24 (14%) |
|  | Polyphagous | 675 (13%) | 12 (7%) |
|  | Detritivores and others | 208 (3.9%) | 21 (12%) |
|  | Adult nectar feeders (grass hostplant) | 1543 (29%) | 9 (5.3%) |

Table S6. Likelihood Ratio Test results for abundance models (GLMMs). Significant effects (p < 0.05) are in bold.

| Method | Response | Parameter | Likelihood ratio test statistic (x2) | p-value |
| --- | --- | --- | --- | --- |
| Traps | All moths | **Treatment** | **48.143** | **<0.001** |
|  |  | **Woody boundary** | **8.309** | **0.004** |
|  |  | Long grass 50m | 0.350 | 0.554 |
|  | Woody feeders | Treatment | 5.254 | 0.072 |
|  |  | **Woody boundary** | **13.387** | **<0.001** |
|  |  | Long grass 50m | 1.385 | 0.239 |
|  | Grass feeders | Treatment | 2.942 | 0.230 |
|  |  | Woody boundary | 1.045 | 0.307 |
|  |  | Long grass 50m | 0.174 | 0.677 |
|  | Unsown forb feeders | **Treatment** | **22.919** | **<0.001** |
|  |  | Woody boundary | 3.041 | 0.081 |
|  |  | Long grass 50m | 0.318 | 0.573 |
|  | Sown forb feeders | **Treatment:Year** | **13.884** | **<0.001** |
|  |  | Woody boundary | 0.249 | 0.618 |
|  |  | Long grass 50m | 0.134 | 0.714 |
|  | Polyphagous | **Treatment** | **11.568** | **0.003** |
|  |  | **Woody boundary** | **18.382** | **<0.001** |
|  |  | **Long grass 50m** | **9.199** | **0.002** |
|  | Detritivores/ other | Treatment | 1.144 | 0.564 |
|  |  | **Woody boundary** | **28.312** | **<0.001** |
|  |  | Long grass 50m | 0.479 | 0.489 |
|  | Adult nectar feeders (grass hostplant) | Treatment | 2.594 | 0.273 |
|  |  | Woody boundary | 0.373 | 0.541 |
|  |  | Long grass 50m | 0.387 | 0.534 |
| Transects | All moths | **Treatment:Year** | **6.291** | **0.043** |
|  |  | Woody 100m | 1.119 | 0.290 |
|  |  | Long grass 25m | 1.374 | 0.241 |
|  |  | **Temperature** | **14.782** | **<0.001** |
|  |  | **Temperature squared** | **12.021** | **<0.001** |
|  | Woody feeders | **Treatment** | **7.250** | **0.027** |
|  |  | **Woody 100m** | **4.945** | **0.026** |
|  |  | Long grass 25m | 0.133 | 0.716 |
|  |  | **Temperature** | **4.979** | **0.026** |
|  |  | **Temperature squared** | **4.810** | **0.028** |
|  | Grass feeders | **Treatment:Year** | **10.991** | **0.004** |
|  |  | Woody 100m | 1.863 | 0.172 |
|  |  | Long grass 25m | 0.186 | 0.666 |
|  |  | Temperature | 1.338 | 0.247 |
|  |  | Temperature squared | 0.975 | 0.323 |
|  | Unsown forb feeders | **Treatment** | **11.779** | **0.003** |
|  |  | Woody 100m | 1.334 | 0.248 |
|  |  | Long grass 25m | 0.588 | 0.443 |
|  |  | **Temperature** | **4.981** | **0.026** |
|  |  | **Temperature squared** | **4.211** | **0.040** |
|  | Sown forb feeders | **Treatment:Year** | **10.006** | **0.007** |
|  |  | Woody 100m | 0.005 | 0.945 |
|  |  | Long grass 25m | 1.873 | 0.171 |
|  |  | Temperature | 0.890 | 0.346 |
|  |  | Temperature squared | 0.333 | 0.564 |
|  | Polyphagous | Treatment | 2.010 | 0.366 |
|  |  | Woody 100m | 0.483 | 0.487 |
|  |  | Long grass 25m | 2.892 | 0.089 |
|  |  | Temperature | 1.239 | 0.266 |
|  |  | Temperature squared | 1.202 | 0.273 |
|  | Detritivores/other | **Treatment** | **11.748** | **0.003** |
|  |  | **Woody 100m** | **9.946** | **<0.001** |
|  |  | Long grass 25m | 2.979 | 0.084 |
|  |  | **Temperature** | **6.947** | **0.008** |
|  |  | Temperature squared | NA | NA |
|  | Adult nectar feeders (grass hostplant) | **Treatment:Year** | **12.143** | **0.002** |
|  |  | Woody 100m | 1.714 | 0.190 |
|  |  | Long grass 25m | 0.034 | 0.854 |
|  |  | Temperature | 0.288 | 0.592 |
|  |  | Temperature squared | 0.002 | 0.965 |

Table S7. Model parameters from abundance models (GLMMs). Treatments: GR = grass only, BC = grass enriched with moth-pollinated flowers, WF = grass and wildflower. Significant effects (p < 0.05) are in bold.

| Method | Response | Parameter | Estimate | Std. error | Z | p-value |
| --- | --- | --- | --- | --- | --- | --- |
| Traps | All moths | **Intercept** | **2.172** | **0.162** | **13.381** | **<0.001** |
|  |  | Treatment BC | 0.056 | 0.049 | 1.136 | 0.256 |
|  |  | **Treatment WF** | **0.313** | **0.048** | **6.468** | **<0.001** |
|  |  | **Woody boundary** | **0.263** | **0.092** | **2.851** | **0.004** |
|  |  | Long grass 50m | 0.038 | 0.065 | 0.576 | 0.565 |
|  | Woody feeders | **Intercept** | **-1.806** | **0.307** | **-5.874** | **<0.001** |
|  |  | **Treatment BC** | **-0.276** | **0.127** | **-2.179** | **0.029** |
|  |  | Treatment WF | -0.052 | 0.126 | -0.415 | 0.678 |
|  |  | **Woody boundary** | **1.020** | **0.240** | **4.259** | **<0.001** |
|  |  | Long grass 50m | -0.175 | 0.147 | -1.190 | 0.234 |
|  | Grass feeders | **Intercept** | **1.239** | **0.202** | **6.121** | **<0.001** |
|  |  | Treatment BC | -0.070 | 0.067 | -1.042 | 0.297 |
|  |  | Treatment WF | 0.045 | 0.066 | 0.686 | 0.493 |
|  |  | Woody boundary | -0.138 | 0.138 | -0.997 | 0.319 |
|  |  | Long grass 50m | 0.040 | 0.099 | 0.408 | 0.683 |
|  | Unsown forb feeders | **Intercept** | **0.761** | **0.175** | **4.358** | **<0.001** |
|  |  | **Treatment BC** | **0.201** | **0.076** | **2.661** | **0.008** |
|  |  | **Treatment WF** | **0.359** | **0.075** | **4.773** | **<0.001** |
|  |  | Woody boundary | 0.207 | 0.133 | 1.559 | 0.119 |
|  |  | Long grass 50m | 0.040 | 0.074 | 0.550 | 0.583 |
|  | Sown forb feeders | **Intercept** | **-1.352** | **0.379** | **-3.570** | **<0.001** |
|  |  | Treatment BC | -0.020 | 0.177 | -0.114 | 0.909 |
|  |  | **Treatment WF** | **1.166** | **0.161** | **7.254** | **<0.001** |
|  |  | Year 2019 | -0.432 | 0.488 | -0.885 | 0.376 |
|  |  | Woody boundary | 0.105 | 0.213 | 0.493 | 0.622 |
|  |  | Long grass 50m | 0.049 | 0.137 | 0.359 | 0.720 |
|  |  | Treatment BC: Year 2019 | 0.008 | 0.288 | 0.027 | 0.978 |
|  |  | **Treatment WF: Year 2019** | **0.751** | **0.247** | **3.036** | **0.002** |
|  | Polyphagous | **Intercept** | **-0.418** | **0.199** | **-2.107** | **0.035** |
|  |  | Treatment BC | 0.159 | 0.093 | 1.711 | 0.087 |
|  |  | **Treatment WF** | **0.310** | **0.091** | **3.393** | **<0.001** |
|  |  | **Woody boundary** | **0.393** | **0.087** | **4.503** | **<0.001** |
|  |  | **Long grass 50m** | **0.153** | **0.050** | **3.057** | **0.002** |
|  | Detritivores and others | **Intercept** | **-1.627** | **0.353** | **-4.606** | **<0.001** |
|  |  | Treatment BC | 0.055 | 0.110 | 0.498 | 0.618 |
|  |  | Treatment WF | 0.118 | 0.111 | 1.068 | 0.285 |
|  |  | **Woody boundary** | **0.951** | **0.186** | **5.124** | **<0.001** |
|  |  | Long grass 50m | -0.078 | 0.112 | -0.693 | 0.488 |
|  | Adult nectar feeders (grass hostplant) | **Intercept** | **0.598** | **0.224** | **2.671** | **0.008** |
|  |  | Treatment BC | -0.009 | 0.081 | -0.116 | 0.908 |
|  |  | Treatment WF | 0.106 | 0.079 | 1.342 | 0.180 |
|  |  | Woody boundary | -0.097 | 0.161 | -0.603 | 0.546 |
|  |  | Long grass 50m | 0.072 | 0.120 | 0.603 | 0.547 |
| Transects | Overall abundance | **Intercept** | **2.207** | **0.159** | **13.861** | **<0.001** |
|  |  | Treatment BC | -0.202 | 0.116 | -1.736 | 0.083 |
|  |  | **Treatment WF** | **0.267** | **0.112** | **2.384** | **0.017** |
|  |  | **Year 2019** | **0.862** | **0.212** | **4.060** | **<0.001** |
|  |  | Woody 100m | 0.054 | 0.052 | 1.040 | 0.298 |
|  |  | Long grass 25m | 0.070 | 0.062 | 1.134 | 0.257 |
|  |  | **Temp** | **1.623** | **0.417** | **3.896** | **<0.001** |
|  |  | **Temp squared** | **-1.473** | **0.419** | **-3.514** | **<0.001** |
|  |  | Treatment BC: Year 2019 | 0.115 | 0.152 | 0.758 | 0.449 |
|  |  | Treatment WF: Year 2019 | -0.253 | 0.149 | -1.697 | 0.090 |
|  | Woody feeders abundance | **Intercept** | **-2.332** | **0.444** | **-5.250** | **<0.001** |
|  |  | **Treatment BC** | **-0.733** | **0.310** | **-2.367** | **0.018** |
|  |  | Treatment WF | -0.0535 | 0.261 | -0.205 | 0.838 |
|  |  | **Woody 100m** | **0.633** | **0.281** | **2.251** | **0.024** |
|  |  | Long grass 25m | 0.123 | 0.336 | 0.367 | 0.714 |
|  |  | **Temperature** | **3.528** | **1.326** | **2.661** | **0.008** |
|  |  | **Temp squared** | **-3.599** | **1.383** | **-2.601** | **0.009** |
|  | Grass feeders abundance | **Intercept** | **0.941** | **0.349** | **2.695** | **<0.001** |
|  |  | **Treatment BC** | **-0.430** | **0.173** | **-2.488** | **0.013** |
|  |  | Treatment WF | -0.244 | 0.168 | -1.454 | 0.146 |
|  |  | **Year 2019** | **1.328** | **0.471** | **2.820** | **0.005** |
|  |  | Woody 100m | 0.111 | 0.083 | 1.338 | 0.181 |
|  |  | Long grass 25m | -0.046 | 0.105 | -0.438 | 0.661 |
|  |  | Temperature | 0.696 | 0.600 | 1.161 | 0.246 |
|  |  | Temp squared | -0.595 | 0.600 | -0.991 | 0.322 |
|  |  | Treatment BC: Year 2019 | 0.202 | 0.216 | 0.937 | 0.349 |
|  |  | **Treatment WF: Year 2019** | **0.508** | **0.215** | **-2.365** | **0.018** |
|  | Unsown forb feeders abundance | **Intercept** | **0.5511** | **0.153** | **3.603** | **<0.001** |
|  |  | Treatment BC | -0.003 | 0.134 | -0.021 | 0.983 |
|  |  | **Treatment WF** | **0.372** | **0.128** | **2.910** | **0.004** |
|  |  | Woody 100m | 0.092 | 0.080 | 1.139 | 0.255 |
|  |  | Long grass 25m | 0.067 | 0.089 | 0.759 | 0.448 |
|  |  | **Temperature** | **1.484** | **0.672** | **2.208** | **0.027** |
|  |  | **Temperature squared** | **-1.374** | **0.677** | **-2.028** | **0.043** |
|  | Sown feeders abundance | **Intercept** | **-3.421** | **0.538** | **-6.363** | **<0.001** |
|  |  | Treatment BC | 0.657 | 0.507 | 1.296 | 0.195 |
|  |  | **Treatment WF** | **2.283** | **0.443** | **5.150** | **<0.001** |
|  |  | Year 2019 | 0.509 | 0.679 | 0.750 | 0.453 |
|  |  | Woody 100m | -0.007 | 0.162 | -0.044 | 0.965 |
|  |  | Long grass 25m | 0.221 | 0.162 | 1.362 | 0.173 |
|  |  | Temperature | 1.075 | 1.141 | 0.942 | 0.346 |
|  |  | Temperature squared | -0.671 | 1.168 | -0.575 | 0.566 |
|  |  | Treatment BC: Year 2019 | -0.589 | 0.699 | -0.843 | 0.399 |
|  |  | Treatment WF: Year 2019 | 0.971 | 0.581 | 1.672 | 0.094 |
|  | Polyphagous | **Intercept** | **-0.774** | **0.352** | **-2.201** | **<0.001** |
|  |  | Treatment BC | 0.001 | 0.168 | 0.007 | 0.994 |
|  |  | Treatment WF | 0.204 | 0.165 | 1.230 | 0.219 |
|  |  | Woody 100m | 0.077 | 0.110 | 0.705 | 0.481 |
|  |  | Long grass 25m | 0.223 | 0.132 | 1.684 | 0.092 |
|  |  | Temp | 1.310 | 1.202 | 1.090 | 0.276 |
|  |  | Temperature squared | -1.353 | 1.265 | -1.070 | 0.285 |
|  | Detritivores and others | **Intercept** | **-2.423** | **0.383** | **-6.323** | **<0.001** |
|  |  | Treatment BC | 0.267 | 0.345 | 0.775 | 0.439 |
|  |  | **Treatment WF** | **0.978** | **0.315** | **3.109** | **0.002** |
|  |  | **Woody 100m** | **0.539** | **0.181** | **2.982** | **<0.001** |
|  |  | Long grass 25m | 0.279 | 0.164 | 1.699 | 0.089 |
|  |  | **Temp** | **0.5112** | **0.192** | **2.657** | **0.008** |
|  |  | Temperature squared | NA | NA | NA | NA |
|  | Nectar feeders abundance (grass hostplant) | Intercept | -0.3266 | 0.387 | -0.843 | 0.399 |
|  |  | Treatment BC | -0.369 | 0.226 | -1.635 | 0.102 |
|  |  | Treatment WF | -0.113 | 0.217 | -0.520 | 0.603 |
|  |  | **Year 2019** | **1.670** | **0.497** | **3.419** | **<0.001** |
|  |  | Woody 100m | 0.143 | 0.111 | 1.284 | 0.199 |
|  |  | Long grass 25m | 0.026 | 0.146 | 0.181 | 0.857 |
|  |  | Temperature | 0.382 | 0.712 | 0.536 | 0.592 |
|  |  | Temperature squared | -0.028 | 0.727 | -0.038 | 0.967 |
|  |  | Treatment BC: Year 2019 | 0.152 | 0.268 | 0.567 | 0.571 |
|  |  | **Treatment WF: Year 2019** | **-0.761** | **0.269** | **-2.829** | **0.005** |

Table S8. Post hoc tests from abundance models (GLMMs) in which there was a significant treatment effect. Treatments: GR = grass only, BC = grass enriched with moth-pollinated flowers, WF = grass and wildflower. Significant effects (p < 0.05) are in bold.

| Method | Response | Contrast | z-ratio | p-value |
| --- | --- | --- | --- | --- |
| Traps | All moths | GR - BC | -1.136 | 0.492 |
|  |  | **GR - WF** | **-6.468** | **<.0001** |
|  |  | **BC - WF** | **-5.257** | **<.0001** |
|  | Unsown forb feeders | **GR - BC** | **-2.661** | **0.021** |
|  |  | **GR - WF** | **-4.773** | **<.0001** |
|  |  | BC - WF | -2.126 | 0.085 |
|  | Sown forb feeders (2018) | GR - BC | 0.114 | 0.993 |
|  |  | **GR - WF** | **-7.254** | **<.0001** |
|  |  | **BC - WF** | **-7.337** | **<.0001** |
|  | Sown forb feeders (2019) | GR - BC | 0.054 | 0.998 |
|  |  | **GR - WF** | **-10.148** | **<.0001** |
|  |  | **BC - WF** | **-10.142** | **<.0001** |
|  | Polyphagous | GR - BC | -1.711 | 0.201 |
|  |  | **GR - WF** | **-3.393** | **0.002** |
|  |  | BC - WF | -1.683 | 0.212 |
| Transects | All moths (2018) | GR - BC | 1.736 | 0.192 |
|  |  | **GR - WF** | **-2.384** | **0.045** |
|  |  | **BC - WF** | **-4.102** | **0.0001** |
|  | All moths (2019) | GR - BC | 0.889 | 0.647 |
|  |  | GR - WF | -0.152 | 0.987 |
|  |  | BC - WF | -1.032 | 0.557 |
|  | Woody feeders | GR - BC | 2.367 | 0.047 |
|  |  | GR - WF | 0.205 | 0.977 |
|  |  | BC - WF | -2.223 | 0.067 |
|  | Grass feeders (2018) | **GR - BC** | **2.489** | **0.034** |
|  |  | GR - WF | 1.454 | 0.313 |
|  |  | BC - WF | -1.066 | 0.535 |
|  | Grass feeders (2019) | GR - BC | 1.770 | 0.180 |
|  |  | **GR - WF** | **5.587** | **<.0001** |
|  |  | **BC - WF** | **3.846** | **<.0001** |
|  | Unsown forb feeders | GR - BC | 0.021 | 1.000 |
|  |  | **GR - WF** | **-2.910** | **0.010** |
|  |  | **BC - WF** | **-2.931** | **0.010** |
|  | Sown forb feeders (2018) | GR - BC | -1.296 | 0.398 |
|  |  | **GR - WF** | **-5.150** | **<.0001** |
|  |  | **BC - WF** | **-4.342** | **<.0001** |
|  | Sown forb feeders (2019) | GR - BC | -0.141 | 0.989 |
|  |  | **GR - WF** | **-8.645** | **<.0001** |
|  |  | **BC - WF** | **-8.676** | **<.0001** |
|  | Non-plant feeders | GR - BC | -0.775 | 0.719 |
|  |  | **GR - WF** | **-3.109** | **0.005** |
|  |  | **BC - WF** | **-2.397** | **0.044** |
|  | Adult nectar feeders (grass hostplant) 2018 | GR - BC | 1.635 | 0.231 |
|  |  | GR - WF | 0.520 | 0.862 |
|  |  | BC - WF | -1.108 | 0.509 |
|  | Adult nectar feeders (grass hostplant) 2019 | GR - BC | 1.472 | 0.304 |
|  |  | **GR - WF** | **5.496** | **<.0001** |
|  |  | **BC - WF** | **4.060** | **<.0001** |

Table S9. Spatial scale selection of surrounding habitat variables using AICc for abundance models (GLMMs). Models are shown in order of descending AICc score. The delta-AICc score is the difference between the AICc for each model and the lowest AICc.

| Response variable | Woody variable | Long grass variable | AICc | delta-AICc |
| --- | --- | --- | --- | --- |
| Moth abundance in traps | Woody boundary | Long grass 50m | 4805.877 | 0 |
|  | Woody boundary | Long grass 100m | 4805.947 | 0.070 |
|  | Woody boundary | Long grass 25m | 4806.175 | 0.298 |
|  | Woody 50m | Long grass 100m | 4811.961 | 6.084 |
|  | Woody 50m | Long grass 25m | 4811.964 | 6.087 |
|  | Woody 100m | Long grass 25m | 4812.157 | 6.280 |
|  | Woody 100m | Long grass 100m | 4812.272 | 6.395 |
|  | Woody 25m | Long grass 25m | 4812.456 | 6.579 |
|  | Woody 25m | Long grass 100m | 4812.838 | 6.961 |
|  | Woody 50m | Long grass 50m | 4812.839 | 6.962 |
|  | Woody 100m | Long grass 50m | 4813.544 | 7.667 |
|  | Woody 25m | Long grass 50m | 4813.674 | 7.797 |
| Moth abundance in transects | Woody 100m | Long grass 25m | 3272.277 | 0 |
|  | Woody 100m | Long grass 100m | 3272.637 | 0.360 |
|  | Woody 100m | Long grass 50m | 3272.747 | 0.470 |
|  | Woody 50m | Long grass 25m | 3273.005 | 0.728 |
|  | Woody boundary | Long grass 100m | 3273.174 | 0.897 |
|  | Woody 25m | Long grass 100m | 3273.202 | 0.925 |
|  | Woody 50m | Long grass 100m | 3273.218 | 0.941 |
|  | Woody boundary | Long grass 25m | 3273.257 | 0.980 |
|  | Woody 25m | Long grass 25m | 3273.274 | 0.997 |
|  | Woody 50m | Long grass 50m | 3273.432 | 1.155 |
|  | Woody boundary | Long grass 50m | 3273.544 | 1.267 |
|  | Woody 25m | Long grass 50m | 3273.563 | 1.286 |

Table S10. Likelihood Ratio Test results for species richness and diversity models (GLMMs). Significant effects (p < 0.05) are in bold.

| Method | Response | Parameter | Likelihood ratio test statistic (x2) | p-value |
| --- | --- | --- | --- | --- |
| Traps | Richness | **Treatment** | **29.311** | **<0.001** |
|  |  | **Woody boundary** | **41.156** | **<0.001** |
|  |  | **Long grass 100m** | **4.637** | **0.031** |
|  | Shannon diversity | **Treatment** | **16.850** | **<0.001** |
|  |  | **Woody boundary** | **32.022** | **<0.001** |
|  |  | **Long grass 100m** | **13.728** | **<0.001** |
| Transects | Richness | **Woody boundary** | **11.7472** | **<0.001** |
|  |  | **Long grass 100m** | **5.1356** | **0.023** |
|  |  | **Treatment:Year** | **8.4035** | **0.015** |
|  | Diversity | **Woody boundary** | **13.9991** | **<0.001** |
|  |  | **Long grass 100m** | **4.9347** | **0.026** |
|  |  | **Treatment:Year** | **12.8735** | **0.002** |

Table S11. Model parameters from abundance models (GLMMs). Treatments: GR = grass only, BC = grass enriched with moth-pollinated flowers, WF = grass and wildflower. P-values are not presented here. To see if main effects are significant, refer to Table S10 and to see if richness/diversity differs significantly between treatment levels, refer to table S12.

| Method | Response | Parameter | Estimate | Std. error | t-value |
| --- | --- | --- | --- | --- | --- |
| Traps | Richness | Intercept | 48.522 | 3.185 | 15.236 |
|  |  | Treatment BC | 4.116 | 2.178 | 1.890 |
|  |  | Treatment WF | 14.446 | 2.181 | 6.623 |
|  |  | Woody boundary | 28.671 | 3.632 | 7.894 |
|  |  | Long grass 100 m | 4.574 | 2.174 | 2.104 |
|  | Shannon diversity | Intercept | 21.805 | 1.665 | 13.099 |
|  |  | Treatment BC | 1.497 | 1.771 | 0.845 |
|  |  | Treatment WF | 7.646 | 1.772 | 4.314 |
|  |  | Woody boundary | 13.536 | 1.874 | 7.222 |
|  |  | Long grass 100 m | 3.934 | 0.978 | 4.024 |
| Transects | Richness | Intercept | 10.298 | 1.051 | 9.798 |
|  |  | Treatment BC | -0.940 | 1.200 | -0.783 |
|  |  | Treatment WF | 3.897 | 1.200 | 3.248 |
|  |  | Year 2019 | 1.733 | 1.199 | 1.446 |
|  |  | Woody boundary | 3.631 | 0.997 | 3.641 |
|  |  | Long grass 100 m | 1.165 | 0.527 | 2.208 |
|  |  | Treatment BC:Year 2019 | 1.667 | 1.696 | 0.983 |
|  |  | Treatment WF:Year 2019 | 4.800 | 1.696 | 2.831 |
|  | Shannon diversity | Intercept | 6.938 | 0.784 | 8.847 |
|  |  | Treatment BC | 0.017 | 0.998 | 0.017 |
|  |  | Treatment WF | 3.242 | 0.998 | 3.249 |
|  |  | Year 2019 | -3.114 | 0.998 | -3.121 |
|  |  | Woody boundary | 2.575 | 0.649 | 3.967 |
|  |  | Long grass 100 m | 0.723 | 0.330 | 2.188 |
|  |  | Treatment BC:Year 2019 | 1.125 | 1.411 | 0.798 |
|  |  | Treatment WF:Year 2019 | 4.870 | 1.411 | 3.452 |

Table S12. Post hoc tests from richness and diversity models (GLMMs) in which there was a significant treatment effect. Treatments: GR = grass only, BC = grass enriched with moth-pollinated flowers, WF = grass and wildflower. Significant effects (p < 0.05) are in bold.

| Method | Response | Contrast | t-ratio | p-value |
| --- | --- | --- | --- | --- |
| Traps | Richness | GR – BC | -1.890 | 0.1611 |
|  |  | GR - WF | -6.622 | <.0001 |
|  |  | BC - WF | -4.700 | 0.0002 |
|  | Shannon diversity | GR – BC | -0.845 | 0.6786 |
|  |  | GR - WF | -4.314 | 0.0005 |
|  |  | BC - WF | -3.461 | 0.0048 |
| Transects | Richness (2018) | GR – BC | 0.783 | 0.7145 |
|  |  | GR - WF | -3.248 | 0.0050 |
|  |  | BC - WF | -4.025 | 0.0004 |
|  | Richness (2019) | GR – BC | -0.606 | 0.8172 |
|  |  | GR - WF | -7.248 | <.0001 |
|  |  | BC - WF | -6.633 | <.0001 |
|  | Diversity (2018) | GR – BC | -0.017 | 0.9998 |
|  |  | GR - WF | -3.249 | 0.0050 |
|  |  | BC - WF | -3.229 | 0.0053 |
|  | Diversity (2019) | GR – BC | -1.145 | 0.4898 |
|  |  | GR - WF | -8.128 | <.0001 |
|  |  | BC - WF | -6.978 | <.0001 |

Table S13. Spatial scale selection of surrounding habitat variables using AICc for species richness models (GLMMs) for traps and transects. Models are shown in order of descending AICc score. The delta-AICc score is the difference between the AICc for each model and the lowest AICc.

| Response variable | Woody variable | Long grass variable | AICc | delta-AICc |
| --- | --- | --- | --- | --- |
| Species richness (traps) | Woody boundary | Long grass_100m | 312.071 | 0 |
|  | Woody boundary | Long grass 50m | 313.453 | 1.383 |
|  | Woody boundary | Long grass 25m | 315.194 | 3.123 |
|  | Woody 50m | Long grass 100m | 335.106 | 23.035 |
|  | Woody 50m | Long grass 50m | 335.198 | 23.128 |
|  | Woody 50m | Long grass 25m | 336.399 | 24.328 |
|  | Woody 25m | Long grass 100m | 344.376 | 32.305 |
|  | Woody 100m | Long gras 100m | 345.120 | 33.049 |
|  | Woody 100m | Long grass 25m | 345.339 | 33.268 |
|  | Woody 100m | Long grass 50m | 345.410 | 33.339 |
|  | Woody 25m | Long grass 50m | 345.683 | 33.612 |
|  | Woody 25m | Long grass 25m | 346.519 | 34.448 |
|  |  |  |  |  |
| Species richness (transects) | Woody boundary | Long grass 100m | 485.105 | 0 |
|  | Woody boundary | Long grass 25m | 487.162 | 2.057 |
|  | Woody boundary | Long grass 50m | 487.268 | 2.163 |
|  | Woody 50m | Long grass 100m | 497.013 | 11.908 |
|  | Woody 100m | Long grass 100m | 497.422 | 12.317 |
|  | Woody 50m | Long grass 50m | 498.302 | 13.197 |
|  | Woody 50m | Long grass 25m | 498.359 | 13.254 |
|  | Woody 100m | Long grass 25m | 498.557 | 13.452 |
|  | Woody 100m | Long grass 50m | 498.594 | 13.489 |
|  | Woody 25m | Long grass 100m | 499.472 | 14.367 |
|  | Woody 25m | Long grass 25m | 501.357 | 16.252 |
|  | Woody 25m | Long grass 50m | 501.461 | 16.355 |

Table S14. Species for which the treatment effect was significant in at least one year and/or sampling method (traps/transects). The effect sizes for WF and BC treatment are shown. GR treatment was used as the reference level. All values are on the link (log) scale. Effects were considered significant if their 95% confidence intervals did not include zero. Two species traits are shown: ‘Wildflower foodplant’ is whether or not the moth species is known to feed on one of the 13 sown wildflower species in the WF treatment. ‘Found nectaring’ refers to whether or not the species was observed nectaring at any flower/fruit during the study. ‘Total caught’ is the total sample size across the two-year experiment. Species for which a treatment type was significant across both sampling methods or across both years are highlighted in blue.

| **Year** | **Method** | **Common name (where applicable)** | **Scientific name** | **WF effect** | **WF sig** | **BC effect** | **BC sig** | **Wildflower foodplant** | **Found nectaring** | **Total caught** |
| --- | --- | --- | --- | --- | --- | --- | --- | --- | --- | --- |
| 2018 | Traps |  | Cochylimorpha straminea | 3.583 | Sig | 0.008 | Non-sig | y | N | 110 |
|  |  |  | Bucculatrix nigricomella | 3.126 | Sig | -12.053 | Non-sig | y | N | 24 |
|  |  | Hollyhock seed moth | Pexicopia malvella | 2.435 | Sig | 0.816 | Non-sig | y | N | 30 |
|  |  |  | Eudonia pallida | 1.766 | Sig | 1.373 | Non-sig | n | N | 31 |
|  |  |  | Aethes smeathmanniana | 1.430 | Sig | -0.334 | Non-sig | y | N | 48 |
|  |  | Single dotted wave | Idaea dimidiata | 1.245 | Sig | 0.502 | Non-sig | n | Y | 55 |
|  |  | Uncertain | Hoplodrina octogenaria | 1.208 | Sig | 0.104 | Non-sig | n | Y | 37 |
|  |  | Common rustic agg | Mesapamea secalis/didyma | 1.152 | Sig | 0.692 | Non-sig | n | Y | 43 |
|  |  | Riband wave | Idaea aversata | 1.091 | Non-sig | 1.539 | Sig | n | Y | 26 |
|  |  | Mottled rustic | Caradrina morpheus | 0.870 | Sig | 0.434 | Non-sig | n | Y | 87 |
|  |  | Square spot rustic | Xestia xanthographa | 0.512 | Sig | 0.461 | Non-sig | n | Y | 349 |
|  |  | Dingy footman | Eilema griseola | 0.386 | Non-sig | 0.621 | Sig | n | N | 110 |
|  |  |  | Cnephasia longana | -0.933 | Non-sig | -1.604 | Sig | y | N | 16 |
|  | Transects |  | Eucosma cana | 2.034 | Sig | -0.023 | Non-sig | y | Y | 34 |
|  |  | Common plume | Emmelina monodactyla | 1.291 | Sig | 0.317 | Non-sig | n | Y | 27 |
|  |  | White plume | Pterophorus pentadactyla | -0.376 | Non-sig | -2.868 | Sig | n | N | 30 |
|  |  |  | Agriphila tristella | -2.351 | Sig | -1.032 | Non-sig | n | N | 22 |
| 2019  2019 | Traps  Traps |  | Cochylimorpha straminea | 2.635 | Sig | -0.178 | Non-sig | y | N | 93 |
|  |  |  | Eucosma cana | 2.593 | Sig | 0.171 | Non-sig | y | Y | 78 |
|  |  |  | Eucosma hohenwartiana | 2.359 | Sig | -0.339 | Non-sig | y | N | 51 |
|  |  |  | Bucculatrix nigricomella | 2.285 | Sig | -12.403 | Non-sig | y | N | 23 |
|  |  | Hollyhock seed moth | Pexicopia malvella | 1.607 | Sig | 0.003 | Non-sig | y | N | 21 |
|  |  | Single dotted wave | Idaea dimidiata | 1.476 | Sig | 0.510 | Non-sig | n | Y | 21 |
|  |  | Mottled rustic | Caradrina morpheus | 1.394 | Sig | 0.481 | Non-sig | n | Y | 20 |
|  |  | Common footman | Eilema lurideola | 1.127 | Sig | 0.444 | Non-sig | n | Y | 37 |
|  |  |  | Celypha lacunana | 0.977 | Sig | -0.029 | Non-sig | n | N | 102 |
|  |  |  | Agriphila geniculea | -0.022 | Non-sig | -1.742 | Sig | n | Y | 35 |
|  |  |  | Agriphila straminella | -0.753 | Sig | -0.329 | Non-sig | n | N | 284 |
|  | Transects |  | Eucosma hohenwartiana | 3.745 | Sig | -12.102 | Non-sig | y | N | 44 |
|  |  | Yarrow plume | Gillmeria pallidactyla | 3.697 | Sig | 1.835 | Non-sig | y | N | 49 |
|  |  |  | Cochylimorpha straminea | 3.567 | Sig | -13.056 | Non-sig | y | N | 123 |
|  |  |  | Endotricha flammealis | 2.662 | Sig | 1.616 | Non-sig | n | Y | 19 |
|  |  |  | Agapeta zoegana | 2.192 | Sig | -12.049 | Non-sig | y | N | 10 |
|  |  |  | Eucosma cana | 1.927 | Sig | -12.735 | Non-sig | y | Y | 15 |
|  |  | Single dotted wave | Idaea dimidiata | 1.888 | Sig | 0.887 | Non-sig | n | Y | 20 |
|  |  | Dwarf cream wave | Idaea fuscovenosa | 1.799 | Non-sig | 2.287 | Sig | n | N | 18 |
|  |  | Silver y | Autographa gamma | -0.024 | Non-sig | 1.666 | Sig | n | Y | 15 |
|  |  |  | Agriphila straminella | -0.527 | Sig | -0.216 | Non-sig | n | N | 846 |
|  |  |  | Crambus perlella | -0.836 | Non-sig | -1.419 | Sig | n | Y | 27 |
|  |  |  | Chrysoteuchia culmella | -1.058 | Sig | -0.279 | Non-sig | n | Y | 1185 |

COULTHARD, E., MCCOLLIN, D. & LITTLEMORE, J. 2016. The use of hedgerows as flight paths by moths in intensive farmland landscapes. *Journal of Insect Conservation,* 20**,** 345-350.

WOIWOD, I. & GOULD, P. 2008. Long-term moth studies at Rothamsted. *The moths of Hertfordshire***,** 31-44.
